# Nonchromatin regulatory functions of the histone variant H2A.B in SWI/SNF genomic deposition

**DOI:** 10.1126/sciadv.adx1568

**Published:** 2025-07-25

**Authors:** Xuanzhao Jiang, Jiayu Wen, Mary L. Nelson, Yasmin Dijkwel, Bradley Cairns, Uta-Maria Bauer, Gene Hart-Smith, Tatiana A. Soboleva, David J. Tremethick

**Affiliations:** ^1^ The John Curtin School of Medical Research, The Australian National University, Canberra, ACT 2601, Australia.; ^2^ Australian Research Council Centre of Excellence for the Mathematical Analysis of Cellular Systems, Canberra, Australia.; ^3^ Howard Hughes Medical Institute, Department of Oncological Sciences, Huntsman Cancer Institute, University of Utah School of Medicine, Salt Lake City, UT 84112, USA.; ^4^ Institute for Molecular Biology and Tumor Research, Philipps-University Marburg, BMFZ, Hans-Meerwein-Str. 2, 35043 Marburg, Germany.; ^5^ Australian Proteome Analysis Facility, Macquarie University, Sydney, NSW 2109, Australia.

## Abstract

The replacement of canonical histones with their variant forms enables the dynamic and context-dependent regulation of the mammalian genome. Histone variants also play key roles in various pathological processes including malignancies. Among these, the aberrant expression of the testis-specific histone variant H2A.B contributes to the pathogenesis of Hodgkin lymphoma. The multifunctionality of histone variants is regulated by their posttranslational modifications (PTMs). However, the PTMs of H2A.B and their functional implications are unknown. Here, we demonstrate that the Amino terminus of H2A.B serves as a central hub for a diverse range of gene regulatory protein-protein interactions, orchestrated by phosphorylation and arginine methylation. This includes a mechanism whereby non–chromatin-bound H2A.B associates with SWI/SNF, which limits its access to the genome. Last, we identify phosphorylated H2A.B as a previously uncharacterized marker of active RNA polymerase II transcription start sites. These findings elucidate a central role for H2A.B in genome regulation and highlight the importance of its PTMs in modulating its multifunctional roles.

## Teaser

The N-terminal tail of H2A.B is an important protein-protein interaction hub mediated by its posttranslational modifications.

## INTRODUCTION

The epigenome encompasses the ensemble of chromatin (CHR)–based and epigenetic modifications that orchestrate gene expression genome-wide. Disruptions to the epigenome, resulting in dysregulated gene expression, are hallmark characteristics of nearly all cancer types (*1*). Despite noteworthy progress, the underlying mechanisms driving these widespread epigenetic aberrations remain poorly understood. Among the various contributors to these alterations, nonallelic variants of canonical histones have gained increasing attention for their contributions to cancer pathogenesis (*2*–*4*). One such histone variant is H2A.B, which is normally expressed in the testis (*5*, *6*). Previously, we and others have shown that H2A.B is aberrantly expressed in certain types of tumors of lymphoid-derived lineages, particularly in Hodgkin lymphoma (HL), which is a common hematopoietic malignancy that affects adults of all ages (*7*, *8*). This cancer is characterized by the presence of mononucleated Hodgkin cells and multinucleated Reed-Sternberg cells, both of which express H2A.B (*8*).

In HL, the unique gene regulatory functions of H2A.B have been hijacked to promote oncogenesis through mechanisms described below (*8*). Notably, several mutations in canonical H2A histones frequently observed in cancer, such as neutralization of the acidic patch on H2A—which destabilizes CHR—occur naturally in H2A.B (*7*). This observation suggests that H2A.B may act as a “preconfigured” oncohistone when aberrantly expressed in inappropriate cellular contexts. Furthermore, another feature of many cancers is the aberrant expression of germ cell–specific genes, commonly referred to as cancer/testis (CT) or cancer/germline (CG) factors (*9*–*12*). These CT factors are thought to modulate signaling pathways or reactivate testis-specific gene expression programs, normally silenced in somatic cells, that contribute to malignant transformation and the development of therapy resistance (*9*–*12*).

Despite the importance of CT factors, mechanistic insights into their functions remain poorly understood, both with respect to their normal roles in gametogenesis and their aberrant contributions to tumor progression. However, studies have suggested that certain CT proteins can act as disruptors of the epigenome and CHR structure, thereby facilitating pathological cellular reprogramming. For instance, BORIS, a germ cell–specific paralog of the CHR architectural protein CTCF, is up-regulated in neuroblastoma (*13*). This aberrant expression promotes the formation of new long-range CHR interactions, resulting in the establishment of superenhancers that drive the expression of oncogenic transcription factors, which ultimately contributes to drug resistance (*13*). Other CT proteins function as readers of acetylated histones. For example, Brdt, a member of the double bromodomain BET family, is aberrantly activated in certain subtypes of lung and breast cancers (*14*). Guided to transcription start sites (TSSs) by histone acetylation, Brdt normally activates key meiotic and postmeiotic genes by recruiting the transcription elongation factor P-TEFb (*14*). Similarly, ATAD2, an adenosine triphosphatase family AAA domain–containing protein that also contains a bromodomain, interacts with acetylated histones H3 and H4 (*15*–*17*). These interactions are thought to enhance CHR accessibility to facilitate transcriptional coactivation of oncogenes and to increase the rate of DNA replication and cancer cell proliferation (*15*–*17*).

H2A.B, encoded on the X-chromosome together with many other CT factors, belongs to the most divergent class of H2A variants, sharing less than 50% amino acid identity with canonical H2A (*18*–*20*). This class, collectively termed “short H2A variants,” also includes H2A.L, H2A.P, and H2A.Q (*18*–*20*). These variants are “short” because they lack the characteristic C-terminal tail found in canonical H2A and are hypothesized to have emerged late in eutherian evolution (*18*–*20*). They are all primarily expressed in the testis, with H2A.B additionally expressed in the brain (*21*). Functional studies reveal that H2A.B plays a unique role in CHR dynamics. In vitro CHR reconstitution assays demonstrate that H2A.B destabilizes nucleosomes and decompacts CHR, facilitating the reversal of CHR-mediated transcriptional repression (*22*, *23*). In vivo, mouse H2A.B (H2A.B.3) exhibits tightly regulated developmental expression, peaking in highly transcriptionally active haploid round spermatids—a stage preceding transcriptional silencing and the replacement of histones with transition proteins (*21*, *22*, *24*). H2A.B.3 enhances transcription and regulates pre-mRNA splicing by being targeted to the TSSs and intron-exon boundaries (*5*, *21*). Notably, both H2A.B and H2A.B.3 function as RNA binding proteins, mediated by their N terminus, further supporting the role of H2A.B in splicing regulation (*18*, *21*).

In HL-derived cell lines, we have demonstrated that H2A.B is co-opted by HL to reprogram the epigenome, thereby altering RNA polymerase (Pol) II transcription and splicing (*8*). Notably, many of the affected genes play pivotal roles in cancer progression. Furthermore, the involvement of H2A.B in HL extends even further by enhancing ribosome biogenesis (*8*). Specifically, H2A.B elevates RNA Pol I transcription levels, showing the capacity of histone variants to adopt new functions in nonphysiological contexts. In addition, H2A.B stimulates the transcription of ribosomal protein genes, further contributing to ribosome biogenesis (*8*). Such an increase in ribosome production is a major characteristic of cancer cells as it supports the elevated growth rates of malignancies (*25*, *26*). Knockdown of H2A.B expression resulted in a marked reduction in HL cell proliferation, emphasizing its essential role in sustaining tumor growth (*8*).

**Fig. 1.**
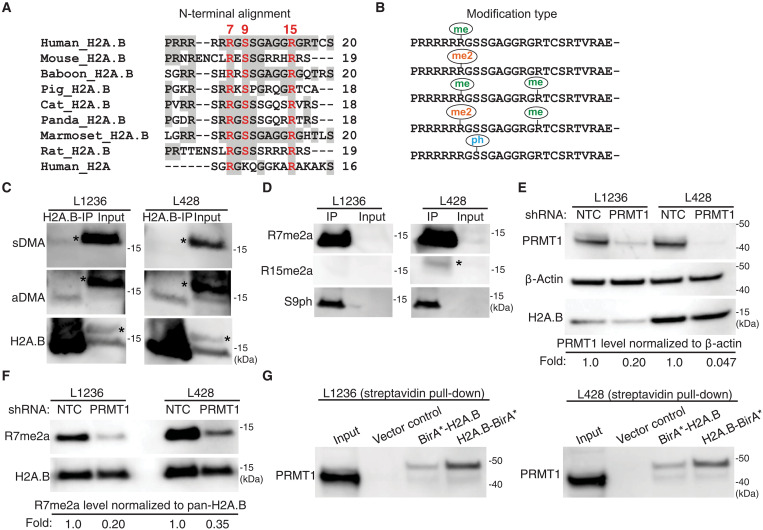
H2A.B undergoes arginine methylation by PRMT1 in HL cells. (**A**) An N-terminal sequence alignment of H2A.B orthologs across various eutherian mammals. The N terminus of human H2A is also shown. Conserved residues R7, S9, and R15 are highlighted in red, while residues identical to human H2A.B are shaded in gray. (**B**) H2A.B PTMs identified in L1236 and L428 cells. These include arginine monomethylation (me), arginine asymmetric dimethylation (me2a), and serine phosphorylation (ph). (**C**) Western blot analyzing H2A.B immunoprecipitates using anti-sDMA and anti-aDMA antibodies. (**D**) Western blot analyzing R7me2a, R15me2a, and S9ph modifications in H2A.B immunoprecipitates using custom-made modification-specific antibodies. (**E**) Western blot analyzing PRMT1 protein levels in nontargeting control (NTC) and PRMT1 shRNA–transduced L1236 and L428 cells. β-Actin served as a loading control. The levels of PRMT1, normalized to β-actin, were quantified and displayed below the blot. (**F**) Western blot analyzing R7me2a levels in H2A.B immunoprecipitates from NTC or PRMT1 shRNA–transduced L1236 and L428 cells. The levels of R7me2a were normalized to total H2A.B and displayed below the blot. (**G**) Proximity-dependent BioID cellular assay confirming that the interaction between PRMT1 and H2A.B. L1236 cells (right panel) and L428 cells (left panel) was transduced with either an empty vector (vector-only), a BirA*-H2A.B vector, or an H2A.B-BirA* vector. Following biotin labeling, streptavidin affinity purification of cell lysates was analyzed by Western blot using an anti-PRMT1 antibody. The positive control was loaded as 1% (v/v) input. The Western blot signal for H2A.B runs below 15 kDa (the molecular weight of H2A.B is 12.7 kDa). The asterisk (*) designates non-H2A.B antibody binding.

Posttranslational modifications (PTMs) of histones and histone variants play a fundamental role in regulating CHR function, often acting by recruiting or excluding effector CHR-binding proteins (*3*, *27*–*29*). However, the PTMs specific to the histone variant H2A.B, and their impact on its function, are not known. Elucidating these modifications is crucial for determining the mechanistic roles of H2A.B in both its normal cellular contexts and aberrant involvement in HL. A defining characteristic of all H2A.B family members is their unstructured, arginine-, serine-, and glycine-rich N-terminal tails (Fig. 1A). These arginine- and glycine-rich motifs are well documented in nucleic acid binding and protein-protein interactions and as substrates for protein arginine methyltransferases (PRMTs) (*30*, *31*).

In this study, we used an integrative approach, combining quantitative proteomics, protein-protein interaction analyses, and functional genomics, to systematically identify the PTMs of H2A.B, investigate their influence on the H2A.B-interacting proteome, and assess their functional impact on genome regulation. Our findings demonstrate that the N-terminal tail of H2A.B serves as an important protein-protein interaction hub, where interactions are modulated by asymmetric arginine dimethylation and serine phosphorylation. We reveal a unique mechanism in which non–CHR-bound H2A.B associates with the human adenosine 5′-triphosphate (ATP)–dependent CHR remodeling complex human SWI/SNF (hSWI/SNF), thereby restricting its access to the genome. This observation establishes an additional layer of genome regulation, which connects histone variants with ATP-dependent CHR remodeling, two CHR-based mechanisms that were previously thought to function independently.

## RESULTS

### H2A.B is arginine methylated by PRMT1 in HL cells

The expression of H2A.B in two HL cell lines (L1236 and L428) provided a tractable cellular system to perform mechanistic studies related to its mode of action in this cancer setting (*8*). One conspicuous feature of H2A.B is its arginine-rich N-terminal tail compared with the lysine-containing N-terminal tail of H2A, which raises the obvious possibility that H2A.B is subject to arginine methylation. An alignment of several mammalian orthologs revealed two conserved arginines (R7 and R15); equivalently conserved residues are also found in human H2A (R3 and R11, respectively) (Fig. 1A). Also clear is a conserved serine amino acid residue (S9), suggesting that phosphorylation may be another important modification of H2A.B (Fig. 1A).

To identify the PTMs of endogenous H2A.B in HL cells, H2A.B was immunoprecipitated from cell lysates using rabbit polyclonal antibodies against H2A.B, and the immunoprecipitates were analyzed by liquid chromatography–tandem mass spectrometry (LC-MS/MS). A total of 95 and 65% of the H2A.B protein sequence was covered by LC-MS/MS analysis in L1236 and L428 cells, respectively, which covered most of the N terminus (fig. S1A). In both cell lines, H2A.B was subjected to monomethylation (fig. S1, B and C) or dimethylation at R7 (R7me and R7me2, respectively) (fig. S1, D and E) and monomethylation at R15 (R15me) (figs. S1, F and G, and S2, A and B). Peptides containing R7me or R7me2 without R15me were identified, but R15me was never detected on its own (Fig. 1B). Phosphorylation of S9 was observed but not in the presence of arginine methylation (fig. S3 and Fig. 1B).

Arginine dimethylation can exist in both symmetric and asymmetric forms (*30*, *31*). To identify the H2A.BR7me2 modification type, we first immunoprecipitated H2A.B and then performed Western blot analysis using pan anti–symmetric dimethyl-arginine (sDMA) or pan anti–asymmetric dimethyl-arginine (aDMA) antibodies (Fig. 1C). The immunoprecipitated H2A.B was clearly recognized by aDMA but not sDMA antibodies. Next, we raised rabbit antibodies against R7me2a, R15me2a (as a control), and S9ph N-terminal peptides. Dot-blot analyses confirmed the high specificity of these antibodies for their respective peptide antigens (fig. S4, A to C). Western blot analysis of H2A.B immunoprecipitates clearly identified asymmetric R7me2 but not asymmetric R15me2 (Fig. 1D). The phosphorylation of S9 was also confirmed (Fig. 1D).

Nine PRMTs exist in mammals (termed PRMT1 to PRMT9). Type 1 PRMTs (PRMT1, PRMT2, PRMT3, PRMT4, PRMT6, and PRMT8) are responsible for aDMA, but PRMT1 is the principal methyltransferase that deposits this PTM in mammalian cells (*30*–*32*). Using a candidate approach, we investigated whether PRMT1 is the writer for H2A.B by establishing a stable PRMT1 short hairpin RNA (shRNA) knockdown system in both L428 and L1236 cells. Greater than 80% knockdown of PRMT1 was achieved (Fig. 1E), which is correlated with a similar level of H2A.BR7me2a decrease (Fig. 1F). On the other hand, as a control, the stable knockdown of PRMT6 only reduced the level of this H2A.B PTM marginally (fig. S5, A and B). Next, we performed H2A.B proximity-dependent biotin identification (BioID) cellular assays to capture native interactions in live cells (*8*). Two BioID H2A.B fusion proteins with the biotin BirA ligase located at the N and C termini, respectively, were stably expressed in L428 and L1236 cells. The level of expression of exogenous H2A.B-BirA* or BirA*-H2A.B protein was comparable to that of endogenous H2A.B (fig. S6A). The biotin-labeled H2A.B-interacting proteins were isolated by streptavidin pull-downs followed by a Western analysis using anti-PRMT1 antibodies.

PRMT1 was clearly observed following streptavidin pull-downs in both L428 and L1236 cells expressing BirA*-H2A.B or H2A.B-BirA* (Fig. 1G). Intriguingly, although PRMT1 in the input was observed as a major ~43-kDa band, a higher molecular protein was observed in the pull-down. This suggests that a minor alternatively spliced variant of PRMT1 rather than the predominant PRMT1 variant is the likely mediator of the asymmetric dimethylation of H2A.BR7 in HL. Alternatively, this higher molecular PRMT1 protein may be the result of PTMs. On the other hand, no PRMT6 protein was observed in streptavidin pull-downs from L428 and L1236 cells expressing BirA*-H2A.B or H2A.B-BirA* (fig. S6B). Last, we performed LC-MS/MS on in vitro–methylated H2A.B-H2B dimers using purified glutathione *S*-transferase (GST)-PRMT1 and identified dimethylation at H2A.BR7 and monomethylation at R15 (fig. S2, C and D). Collectively, these results demonstrate that PRMT1 is the primary writer of the H2A.BR7me2a modification.

### Arginine methylation alters the protein interactome of H2A.B

To understand the consequences of arginine methylation and serine phosphorylation on H2A.B protein-protein interactions, we used a stable isotope labeling by amino acids in cell culture (SILAC) quantitative proteomics approach. For these experiments, L428 cells were cultured in medium supplemented with amino acids containing either normal (“light”), “medium,” or “heavy” stable arginine and lysine isotopes (see Materials and Methods). Nuclear extracts were prepared from each isotope type of labeled cells and then incubated with unmodified or modified (R7me2a or R7me) biotinylated H2A.B N-terminal peptides. Following the isolation of peptide-interacting proteins using streptavidin beads, three samples (“light pull-down,” “medium pull-down,” and “heavy pull-down”) were mixed together in equal proportions as described in table S1 and then analyzed by LC-MS/MS.

The relative abundance of proteins was measured on the basis of the log_2_ ratios of intensities between “heavy” and “light,” “medium” and “light,” and “heavy” and “medium” peptides. This approach was repeated with unmodified and R15me-modified biotinylated H2A.B N-terminal peptides, except that nuclear extracts were prepared from L428 cells grown only in “light” or “heavy” media (table S1) (we note that a double-methylated R7 and R15 peptide was unable to be synthesized). The log_2_ forward ratio and reverse ratio were plotted in a scatter pot (fig. S7). Proteins with a log_2_ forward ratio > 1 and log_2_ reverse ratio < −1 were deemed candidate protein interactors for the modified peptides. Proteins with a log_2_ forward ratio < −1 and log_2_ reverse ratio > 1 were deemed candidate protein readers for the unmodified peptides (fig. S7). Proteins were also identified that bound either to unmodified or modified N-terminal peptides but not to both.

First, we compared protein interaction differences between unmodified versus R7me-modified H2A.B N-terminal tails. No proteins could distinguish between the unmodified N terminus and this PTM (fig. S7, A and B). A similar observation was noted when comparing unmodified and R15me-modified H2A.B N-terminal tails (fig. S7C). In contrast, the unmodified H2A.B tail, as did the R7me-modified tail, exhibited distinct protein interaction partners relative to R7me2a (fig. S7, D to I). Notably, most of these proteins were involved in RNA splicing (tables S2 and S3). This is consistent with our previous studies demonstrating that H2A.B.3 (the mouse ortholog of H2A.B) interacts with splicing proteins and plays an important role in the pre-mRNA splicing process in the mouse testis.

Next, we compared the protein interaction differences between unmodified versus S9ph-modified H2A.B N-terminal tails. The S9ph peptide displayed a different group of interacting proteins compared with the unmodified peptide (fig. S7J); 29 phosphorylation-dependent interacting proteins were identified (tables S4 and S5). The protein candidates were substantially enriched (21%) in Pol I–mediated ribosomal RNA transcription, including two Pol I subunits (POLR1A/RPA194 and POLR1C/RPA40) and four related transcription factors (TAF1D, TAF1C, TTF1, and TCOF1). These results are in complete agreement with our previous study where we demonstrated that H2A.B enhanced ribosomal DNA (rDNA) transcription in L1236 and L428 HL cell lines (*8*). Moreover, we also showed that H2A.B directly interacts with POLR1A/RPA194 by using BioID cellular assays. Intriguingly, S9ph-interacting proteins included components of the HIRA histone chaperone complex (HIRA, UBN1, UBN2, and CABIN; tables S4 and S5).

**Fig. 2.**
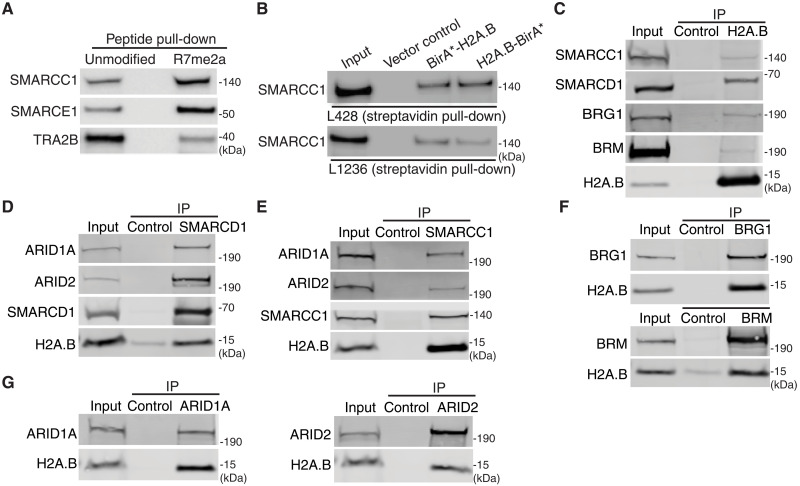
Interaction between hSWI/SNF and H2A.B independent of CHR. (**A**) A Western blot detecting SMARCC1, SMARCE1, and TRA2B binding to unmodified or R7me2a-modified H2A.B peptides. The peptide pull-downs were performed using nuclear extracts from L428 cells. (**B**) BioID assays demonstrating that the proximity of SMARCC1 to H2A.B. L428 cells (upper panel) and L1236 cells (lower panel) were transduced either with an empty vector (vector-only), a BirA*-H2A.B vector, or an H2A.B-BirA* vector. Streptavidin affinity purification of the cell lysate was analyzed by Western blot using an anti-SMARCC1 antibody. (**C**) Co-IP analysis of H2A.B with SMARCC1, SMARCD1, BRG1, and BRM. (**D**) Co-IP analysis of SMARCD1 with H2A.B, ARID1A, and ARID2. (**E**) Co-IP analysis of SMARCC1 with H2A.B, ARID1A, and ARID2. (**F**) Co-IP analysis of BRG1 (upper panel) and BRM (lower panel) with H2A.B. (**G**) Co-IP analysis of SMARCC1 with H2A.B, ARID1A, and ARID2. (F) Co-IP analysis of ARID1A (left panel) or ARID2 (right panel) with H2A.B. All IP experiments were performed using CN fractions. The 1% (v/v) input sample was included as a positive control.

Unexpected, unique H2A.B-protein interaction networks were identified for R7me2a compared with unmodified or R7me-modified H2A.B (48 proteins in total; tables S6 and S7). Fifteen proteins were functionally associated with ribosomal RNA transcription and pre-rDNA processing, which again supports our previous observation that H2A.B is required for ribosome biogenesis in HL (*8*). Notably, eight proteins were detected as known subunits of the human ATP-dependent remodeling complex SWI/SNF (hSWI/SNF; ARID2, SMARCC1, SMARCA2, SMARCC2, PBRM1, BRD7, BRD9, and ACTL6A) (tables S6 and S7). We repeated the N-terminal unmodified and R7me2a peptide pull-down experiments and performed Western blot analyses using antibodies against core subunits of the hSWI/SNF complexes canonical BRG1/BRM–associated factor complex (CBAF) and polybromo-associated BAF complex (PBAF) (SMARCC1 and SMARCE1). The N terminus of H2A.B selectively targeted these core subunits, which was enhanced by the R7me2a modification, which recapitulated the SILAC–LC-MS/MS results (Fig. 2A). On the other hand, the splicing protein TRA2B preferably binds to the unmodified H2A.B tail, also consistent with the results of the SILAC–LC-MS/MS experiments (Fig. 2A). However, an alternative explanation for the preference of hSWI/SNF for R7me2a-modified H2A.B is that while hSWI/SNF can bind the unmodified tail with high affinity, other proteins—including splicing factors (tables S2 and S3)—compete more effectively, reducing its availability for hSWI/SNF interaction (see below and Discussion).

### H2A.B interacts with CBAF and PBAF in HL

A large proportion of H2A.B exists in a non–CHR-bound state [the cytoplasmic/nucleoplasmic (CN) fraction] (*8*), including H2A.BR7me2a (fig. S8A) as well as hSWI/SNF (fig. S8B). This raises the intriguing possibility that if H2A.B is a bona fide hSWI/SNF interacting partner, this contact may occur before their association with CHR. To investigate this hypothesis, we first used the BioID cellular assay to investigate whether H2A.B interacts with hSWI/SNF in living cells. Next, we performed coimmunoprecipitation (co-IP) assays and performed gel filtration chromatography experiments using the CN cellular fraction.

Biotinylated proteins from either BirA*-H2A.B–, H2A.B-BirA*–, or empty vector–transduced L1236 or L428 cells were isolated using streptavidin beads and analyzed by Western blotting using antibodies against the core subunit SMARCC1. SMARCC1 could clearly be detected in both L428 and L1236 cells, supporting the notion that H2A.B and this hSWI/SNF subunit are in close proximity (Fig. 2B).

hSWI/SNF exists as different distinct assemblies with the major complexes being CBAFs and PBAFs (*33*–*36*). CBAF and PBAF share eight common auxiliary subunits including SMARCC1, SMARCE1, and SMARCD1. The two complexes are distinguished by CBAF-specific subunits (ARID1A/B, DPF1/2/3, and SS18) and PBAF-specific subunits (ARID2, PHF10, PBRM1, and BRD7). Next, we performed H2A.B (Fig. 2C), SMARCD1 (Fig. 2D), and SMARCC1 (Fig. 2E) co-IP using the CN fraction prepared from L428 cells. H2A.B can coimmunoprecipitate with SMACC1, SMARCD1, BRG1, and BRM (Fig. 2C). Conversely, SMARCD1 and SMARCC1 can coimmunoprecipitate with H2A.B and with CBAF- and PBAF-specific subunits, as expected (Fig. 2, D and E). Both BRG1 and BRM can also immunoprecipitate H2A.B (Fig. 2F). H2A.B could also be identified in co-IP experiments using antibodies against the CBAF-specific subunit ARID1A and the PBAF-specific subunit ARID2, indicating that H2A.B is associated with both types of hSWI/SNF complexes (Fig. 2G).

It is important to point out that free canonical histones, e.g., H2A and H2B, or other histone variants, e.g., H2A.Z, are barely detectable in this unbound CHR fraction as expected (fig. S8, A and C). Therefore, H2A.B is the only histone that has the opportunity to interact specifically with hSWI/SNF in the CN fraction. Furthermore, H2B does not coimmunoprecipitate with H2A.B, showing that H2A.B does not exist as a dimer with H2B in the CN fraction (fig. S8C).

Given this association between H2A.B and hSWI/SNF, we wondered whether H2A.B and hSWI/SNF may exist as a co-complex. To explore this possibility, we fractionated the HL CN faction using gel filtration. H2A.B cofractionates with BRG1, SMARCC1, and SMARCD1 (fractions 10 to 12) as a high-molecular-weight complex (fig. S9). In conclusion, the H2A.B histone variant is a bona fide interactor of hSWI/SNF in HL and, moreover, this interaction is non-nucleosomal.

### The H2A.B N terminus directly interacts with SWI/SNF in vitro

**Fig. 3.**
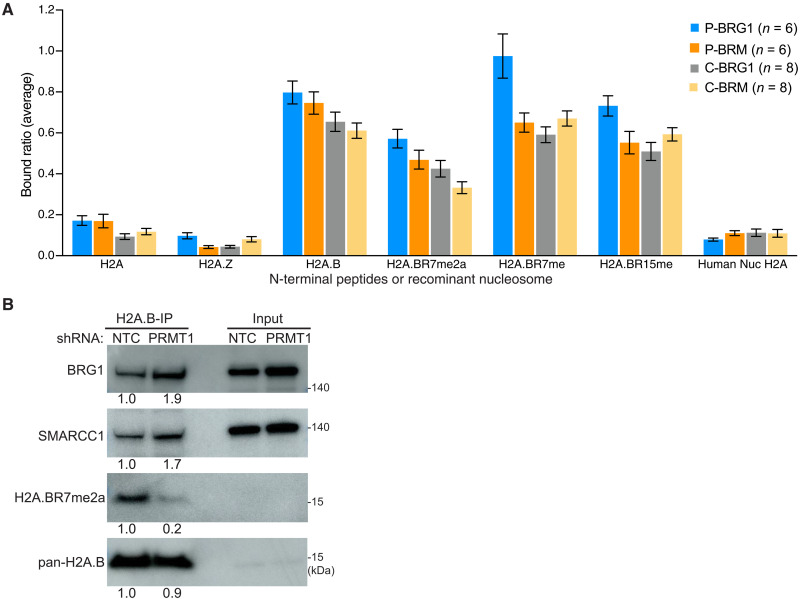
hSWI/SNF preferentially binds to the N-terminal tail of H2A.B. (**A**) H2A, H2A.Z, H2A.B, H2A.BR7me2a, H2A.BR7me, or H2A.BR15me biotin-tagged N-terminal peptides were incubated with purified recombinant CBAF (BRG1 and BRM) or PBAF (BRG1 and BRM). Peptide-bound hSWI/SNF complexes were subjected to a Western blot analysis, and relative peptide binding affinities were determined using a combination of different common and subunit-specific CBAF and PBAF antibodies (see Materials and Methods). As an additional control, biotinylated recombinant human nucleosomes were also examined as an hSWI/SNF binding substrate. Error bars represent the standard error of the mean. (**B**) Western blots analyzing the levels of CN H2A.B coimmunoprecipitated BRG1 and SMARCC1 from NTC or PRMT1 shRNA–transduced L428 cells.

To investigate whether the N terminus of H2A.B can directly interact with SWI/SNF and determine whether R7me2a is directly required, we purified exogenously expressed CBAF (with BRG1 or BRM) and PBAF (with BRG1 or BRM) from Expi293^TM^ cells (fig. S10). Binding reactions contained a biotinylated N-terminal H2A.B peptide (unmodified, R7me2a-modified, R7me-modified, or R15me-modified) with either CBAF or PBAF. We also included the N termini of H2A and H2A.Z as controls in these experiments. Following streptavidin pull-down, bound SWI/SNF complexes were quantified by Western blot analysis using antibodies against BRG1, BRM, common CBAF and PBAF subunits (SMARCC1 and SMARCD1), CBAF-specific subunit (DPF2), and PBAF-specific subunit (PHF10) (fig. S11). Notably, all H2A.B peptides (modified and unmodified) could bind strongly to all SWI/SNF complexes (CBAF with BRG1 or BRM and PBAF with BRG1 or BRM) compared with H2A and H2A.Z (Fig. 3). This supports our hypothesis that the H2A.B N terminus is an interaction partner of hSWI/SNF. Intriguingly, however, of the different H2A.B peptide types, the R7me2a modification reduced BAF and PBAF binding consistently (Fig. 3A). This supports the alternative proposal that hSWI/SNF can bind the unmodified tail with high affinity, but other proteins in the cytoplasm/nucleoplasm compete more effectively.

To investigate this further, we used the stable PRMT1 shRNA knockdown system in L428 HL cells to determine whether the loss of R7 asymmetric dimethylation would affect H2A.B-hSWI/SNF binding negatively or positively (Fig. 3B). Recapitulating the in vitro hSWI/SNF binding observation, the IP of H2A.B from the CN fraction revealed an increase in the coprecipitation of BRG1 and SMARCC1 from PRMT1-depleted CN extracts. Collectively, this argues that SWI/SNF is not a direct reader of R7me2, but this modification may indirectly regulate H2A.B-SWI/SNF protein-protein interactions (see Discussion).

### R7 H2A.B methylation is depleted at the TSS

**Fig. 4.**
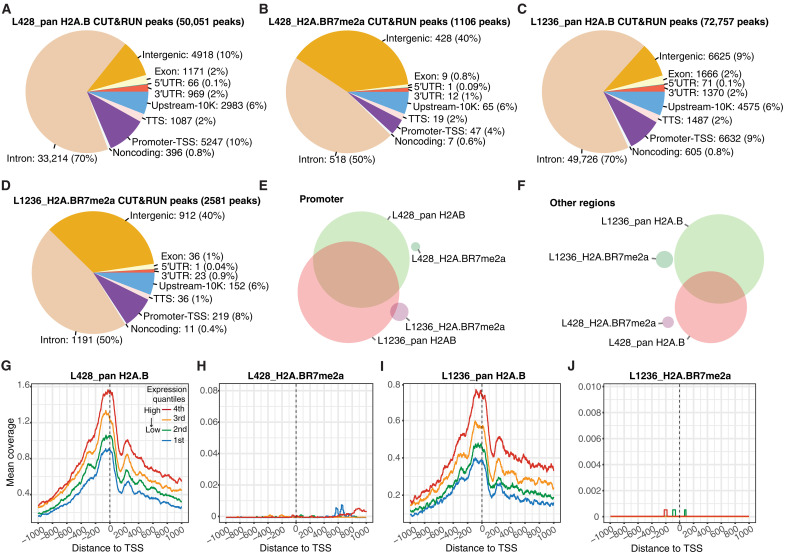
H2A.BR7me2 is not present at the TSS of RNA Pol II genes. Genomic annotations are categorized as follows: upstream-10K, regions encompassing 2 to 10 kb downstream of the TSS; promoter-TSS, regions encompassing 2 kb upstream to 500 bp (base pairs) downstream of the TSS; TTS, regions encompassing 100 bp upstream to 1 kb downstream of the transcription termination site; UTR, untranslated regions. (**A**) Genomic annotation of pan H2A.B CUT&RUN peaks in L428 cells. (**B**) Genomic annotation of H2A.BR7me2a CUT&RUN peaks in L428 cells. (**C**) Genomic annotation of pan H2A.B CUT&RUN peaks in L1236 cells. (**D**) Genomic annotation of H2A.BR7me2a CUT&RUN peaks in L1236 cells. (**E**) Venn diagram displaying the overlap between pan H2A.B and H2A.BR7me2a CUT&RUN peaks in promoter-TSS regions. (**F**) Venn diagram displaying the overlap between pan H2A.B and H2A.BR7me2a CUT&RUN peaks in all other genomic regions. (**G**) H2A.B mean CUT&RUN coverage aligned between −1 and +1 kb from the TSS ranked according to the level of gene expression in L428 cells. (**H**) H2A.BR7me2a mean CUT&RUN coverage aligned between −1 and +1 kb from the TSS ranked according to the level of gene expression in L428 cells. (**I**) H2A.B mean CUT&RUN coverage aligned between −1 and +1 kb from the TSS ranked according to the level of gene expression in L1236 cells. (**J**) H2A.BR7me2a mean CUT&RUN coverage aligned between −1 and +1 kb from the TSS ranked according to the level of gene expression in L1236 cells.

An important location of H2A.B is at the TSS of active genes both in proper physiological contexts and when it is aberrantly expressed in HL (*8*, *18*). To determine whether H2A.BR7me2a occupies the same or distinct genomic locations compared with total H2A.B, we performed H2A.B and H2A.BR7me2a CUT&RUN experiments in L428 and L1236 HL cells. There were fewer H2A.BR7me2a CUT&RUN peaks than pan H2A.B CUT&RUN peaks globally and at all genomic locations. Furthermore, H2A.BR7me2a was relatively more enriched at intergenic regions compared with pan H2A.B (Fig. 4, A to D).

Comparing the promoter overlap of pan H2A.B and H2A.BR7me2a between L428 and L1236 HL cells, a strong intersection was observed with pan H2A.B, indicating that H2A.B occupies common promoters in both cell lines. On the other hand, no such commonality occurs for H2A.BR7me2a. This suggests that H2A.BR7me2a does not play an important role at promoters (Fig. 4E). Likewise, the absence of overlap at other genomic loci suggests that H2A.BR7me2a does not play a specific role in establishing or maintaining the HL phenotype (Fig. 4F). To explore this further, genes transcribed by RNA Pol II were separated into groups according to their expression level (repressed, low, medium, and high). For each gene group, a single line represents the mean coverage at each base pair, which was aligned with the TSS (±1 kb). As shown previously, there was a positive correlation between the appearance of H2A.B at the TSS and increasing levels of transcription (Fig. 4, G and I). Conversely, H2A.BR7me2a is absent from the TSS (Fig. 4, H and J). Collectively, this shows that the arginine methylation of H2A.B is not a mark of active promoters and that H2A.BR7me2a may have a more important function(s) when not bound to CHR. Nevertheless, its relative enrichment at noncoding intergenic regions—compared to pan H2A.B—raises the possibility of additional dynamic genomic functions in this genomic context (Fig. 4, A to D).

### H2A.B antagonizes the genomic deposition of hSWI/SNF

**Fig. 5.**
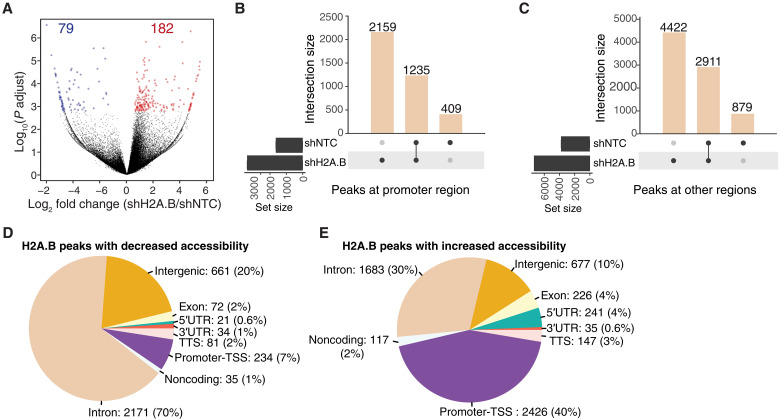
H2A.B antagonizes the genomic deposition of the hSWI/SNF complex. (**A**) Volcano plot of the differential occupancy of BRG1 at H2A.B peaks in L1236 cells transduced with an H2A.B-targeting shRNA (shH2A.B) lentiviral vector normalized to an shNTC. Peaks with altered BRG1 levels (FDR < 0.2) are highlighted (red and blue peaks have an increase or a decrease in BRG1 occupancy, respectively). (**B**) UpSet plots showing individual and shared BRG1 peaks at promoter-TSS regions in shH2A.B- and shNTC-transduced L1236 cells. (**C**) UpSet plots showing individual and shared BRG1 peaks at non–promoter-TSS genomic regions in shH2A.B- and shNTC-transduced L1236 cells (FDR < 0.1). (**D**) Genomic annotation of the H2A.B peaks with decreased ATAC-seq accessibility following H2A.B knockdown (FDR < 0.1). (**E**) Genomic annotation of the H2A.B peaks with increased ATAC-seq accessibility following H2A.B knockdown (FDR < 0.1).

Given that both H2A.B and hSWI/SNF are targeted to the TSSs of active genes (fig. S12, A to C), and they appear to coexist in the same complex (fig. S9), an attractive hypothesis is that H2A.B may guide hSWI/SNF to this important functional element. To test this hypothesis, the genomic enrichment of BRG1 that overlaps with existing H2A.B nucleosomes was investigated in H2A.B-depleted versus control HL cells following peak calling. Unexpectedly, there were more H2A.B nucleosome sites with a modest increase in BRG1 enrichment (182 peaks) compared with those H2A.B locations that lost BRG1 (79 peaks) in H2A.B-depleted L1236 HL cells (Fig. 5A). This result suggests that relatively more BRG1 associates with the genome upon the removal of H2A.B. H2A.B knockdown increased the levels of BRG1 at active TSSs (fig. S12D). Furthermore, only ~0.5% of H2A.B CUT&RUN peaks displayed a differential association with BRG1 after H2A.B knockdown. This argues against the notion that H2A.B escorts hSWI/SNF to the TSS.

To analyze this further, we mapped all BRG1 peaks genome-wide in control and H2A.B knockdown L1236 HL cells and partitioned them into promoter regions (−2 and 2 kb within TSS) and all other genomic regions. Although the location of the 1235 BRG1 peaks (32.5% of all promoter peaks) remained unchanged between the knockdown and control samples, 2159 new peaks of BGR1 (56.7%) were observed after the knockdown of H2A.B (Fig. 5B). A similar trend was observed for all other genomic regions, i.e., upon the loss of H2A.B, there was a marked increase in the genomic deposition of hSWI/SNF (Fig. 5C). Collectively, these results show that H2A.B inhibits the genomic deposition of hSWI/SNF.

An obvious prediction is that following H2A.B knockdown and the marked increase in the deposition of hSWI/SNF at promoters, there will also be a major increase in promoter accessibility. To investigate this prediction, we performed assay for transposase-accessible CHR using sequencing (ATAC-seq) and examined the differential change in accessibility at H2A.B CUT&RUN peaks upon H2A.B knockdown compared with control L1236 HL cells. This revealed remarkable differences in CHR accessibility according to genomic location. The genomic region with reduced accessibility was predominantly annotated to the introns (70% of all H2A.B peaks with a decrease in ATAC-seq accessibility were at introns; Fig. 5D), whereas the genomic region with increased accessibility was predominantly annotated to the promoter-TSS (40% of all H2A.B peaks with an increase in ATAC-seq accessibility were at promoters; Fig. 5E). Together, these results clearly show that H2A.B removal makes CHR less accessible at intronic regions, consistent with its ability to decompact CHR but more accessible at promoters, which is concordant with an increase in hSWI/SNF deposition.

### S9 H2A.B phosphorylation is enriched at the TSS

**Fig. 6.**
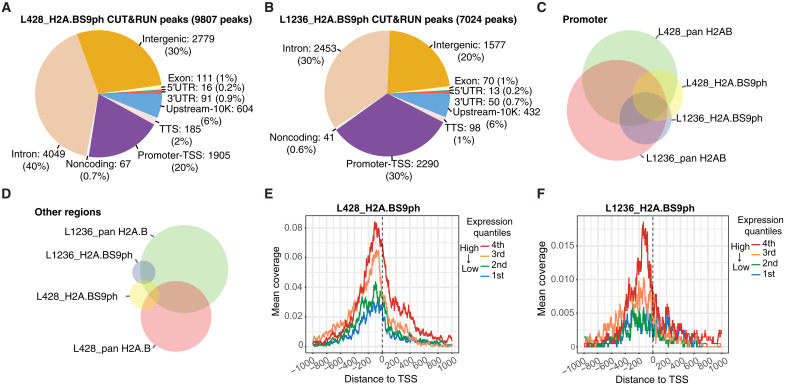
Phosphorylation of H2A.B is correlated with high levels of transcription. (**A**) Genomic annotation of H2A.BS9ph CUT&RUN peaks in L428 cells. (**B**) Genomic annotation of H2A.BS9ph CUT&RUN peaks in L1236 cells. (**C**) Venn diagram displaying the overlap between pan H2A.B and H2A.BS9ph CUT&RUN peaks in promoter-TSS regions. (**D**) Venn diagram displaying the overlap between pan H2A.B and H2A.BS9ph CUT&RUN peaks in non–promoter-TSS genomic regions. (**E**) H2A.BS9ph mean CUT&RUN coverage aligned between −1 and +1 kb from the TSS ranked according to the level of gene expression in L428 cells. (**F**) H2A.BS9ph mean CUT&RUN coverage aligned between −1 and +1 kb from the TSS ranked according to the level of gene expression in L1236 cells.

Next, we investigated whether H2A.BS9ph demarcates different regions of the genome. The genomic annotation profile of H2A.BS9ph differed markedly from that of bulk H2A.B and H2A.BR7me2a. Notably, H2A.BS9ph is two to three times more enriched at promoter regions compared with total H2A.B in L428 (compare Fig. 6A with Fig. 4A) and L1236 HL cells (compare Fig. 6B with Fig. 4C). Furthermore, unlike H2A.BR7me2a, there was a prominent overlap of the presence of H2A.BS9ph on the same promoters between both cell lines (compare Fig. 6C with Fig. 4E). On the other hand, there was no overlap of H2A.BS9ph between L428 and L1236 cells in other regions of the genome (Fig. 6D). Mapping H2A.BS9ph at the TSS showed a strong positive correlation with the level of transcription (Fig. 6, E and F).

Given that promoter regions account for 1 to 2% of the total genome, this analysis therefore reveals a 10- to 30-fold enrichment of H2A.BS9ph at promoters since 20 to 30% of H2A.BS9ph peaks are found at these sites (Fig. 6, A and B). This strong enrichment, coupled with inter–cell line overlap and a positive transcriptional correlation, strongly argues that H2A.BS9ph is a previously unknown PTM associated with active TSSs.

RNA Pol II transcription is broadly repressed during mitosis, a mechanism that safeguards accurate chromosome segregation (*37*) and ensures proper transcriptional control across the cell cycle (*38*). Despite this global suppression, a subset of transcription factors and CHR remodelers retains their association with specific genomic loci, effectively “bookmarking” genes for rapid reactivation in daughter cells (*37*). Given this, we sought to determine whether the histone variant H2A.B—and its phosphorylated form—remains associated with CHR during mitosis or is displaced. To investigate this, we performed immunostaining on asynchronous L428 cells, as well as on cells arrested at the G_2_-M boundary using nocodazole, using antibodies against pan H2A.B and H2A.BS9ph. Under both conditions, neither H2A.B nor H2A.BS9ph was detectable on mitotic chromosomes (fig S13, A to D). These findings suggest that H2A.B is evicted from CHR during mitosis. However, we cannot rule out the possibility that mitotic CHR compaction obscures epitope accessibility, preventing antibody recognition.

### The N terminus of H2A.B regulates the incorporation of H2A.B into CHR

**Fig. 7.**
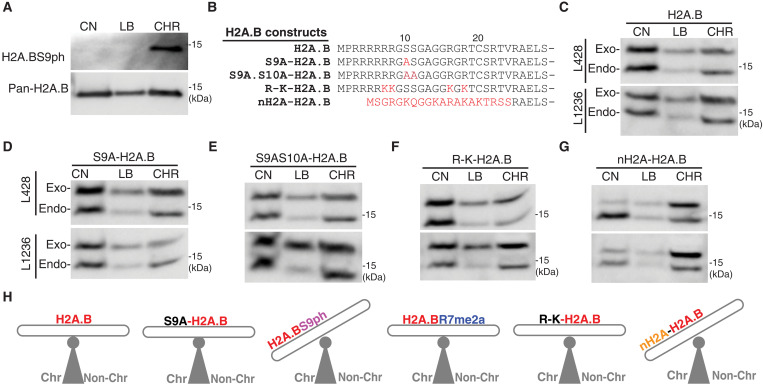
The N terminus of H2A.B regulates its incorporation into CHR. (**A**) Western blot analysis of the subcellular location of H2A.BS9ph. H2A.B was immunoprecipitated from L428 CN, LB, and CHR fractions. The Western blot was then probed with anti-H2A.BS9ph antibodies. (**B**) N-terminal sequences of H2A.B and the different serine-to-alanine and arginine-to-lysine amino acid residue substitutions and the complete replacement of the N terminus of H2A.B, exogenously expressed in HL cell lines. (**C**) Western blot analyses of endogenous H2A.B (Endo) and exogenously expressed wild-type H2A.B construct (Exo) in different subcellular fractions of L428 cells (upper panel) and L1236 cells (lower panel). (**D**) Western blot analyses of endogenous H2A.B (Endo) and exogenously expressed mutant S9A-H2A.B construct (Exo) in different subcellular fractions of L428 cells (upper panel) and L1236 cells (lower panel). (**E**) Western blot analyses of endogenous H2A.B (Endo) and exogenously expressed mutant S9AS10A-H2A.B construct (Exo) in different subcellular fractions of L428 cells (upper panel) and L1236 cells (lower panel). (**F**) Western blot analyses of endogenous H2A.B (Endo) and exogenously expressed mutant R-K-H2A.B construct (Exo) in different subcellular fractions of L428 cells (upper panel) and L1236 cells (lower panel). (**G**) Western blot analyses of endogenous H2A.B (Endo) and exogenously expressed mutant nH2A-H2A.B construct (Exo) in different subcellular fractions of L428 cells (upper panel) and L1236 cells (lower panel). (**H**) Schematic illustrating the effects of N-terminal PTMs and N-terminal mutations on H2A.B CHR incorporation.

Next, we investigated whether the phosphorylation of H2A.B influences the dynamics of H2A.B incorporation into CHR by using the aforementioned KCl-based cellular fractionation method followed by pan H2A.B IP and Western blot analysis using anti-H2A.BS9ph antibodies. Notably, phosphorylation shifted the dynamics of H2A.B CHR incorporation such that H2A.BS9ph was predominantly incorporated into CHR (Fig. 7A). To investigate whether phosphorylation is required for the incorporation of H2A.B into CHR or whether phosphorylation occurs following its deposition into CHR, we exogenously expressed One-Strep–tagged H2A.B mutants in which S9 or S9 and S10 were replaced with alanine amino acid residues (S9A-H2A.B and S9A.S10A-H2A.B; Fig. 7B). In addition, to explore the importance of methylated arginine residues R7 and R15, these amino acid residues, along with R6 and R17, were mutated to lysine residues (R-K-H2A.B; Fig. 7B). Last, we replaced the entire N terminus of H2A.B with the N terminus of H2A (nH2A-H2A.B).

Neither S9A nor the combined S9A and S10A H2A.B mutants prevented the incorporation of H2A.B into CHR, as these mutants displayed the same distribution between the CN fraction and the CHR-bound fraction as endogenous H2A.B and exogenous H2A.B (Fig. 7, C to E). Therefore, given that these H2A.B mutants did not inhibit the incorporation of H2A.B, this suggests that phosphorylation of H2A.B occurs after it is deposited into CHR and that this modification helps to keep it stably bound there. Furthermore, the R-K-H2A.B mutant also did not alter the dynamics of H2A.B incorporation into CHR (Fig. 7F). In notable contrast, the nH2A-H2A.B mutant was largely incorporated into CHR, similar to the phosphorylated form of H2A.B (Fig. 7G). Therefore, the N terminus of H2A.B is necessary for retaining a proportion H2A.B in a soluble non–CHR-associated form. Given that the N terminus of H2A.B can interact with RNA or proteins, this result suggests that the presence of H2A.B in the non–CHR-bound form might be determined by the protein or RNA-interacting partners of H2A.B in the nucleoplasm rather than by its PTMs.

## DISCUSSION

H2A.B plays a pivotal role in modulating the phenotype of HL through multifaceted mechanisms (*8*, *18*). It regulates the HL transcriptome, including the expression of cancer-related genes, by enhancing transcriptional activity and influencing pre-mRNA splicing. In addition, H2A.B promotes ribosome biogenesis, aligning with its critical role in supporting the high proliferation rates of HL-derived L1236 and L428 cells (*8*, *18*). While histones and their variants are functionally regulated by PTMs, the specific PTMs of H2A.B have not been characterized previously. This study aimed to elucidate the PTMs of H2A.B, map their nuclear and genomic distributions, and assess their potential to modulate protein-protein interaction networks and the corresponding impact to genome function. We show here that (i) the N terminus of H2A.B undergoes PTMs, specifically arginine methylation and phosphorylation; (ii) these PTMs enable the N terminus of H2A.B to serve as a multiprotein interaction hub, and most notably, it is non–CHR-bound H2A.B that associates with SWI/SNF; (iii) CHR incorporation of H2A.B is tightly regulated by its N terminus, and moreover, only phosphorylated H2A.B is stably incorporated into CHR; and (iv) phosphorylated H2A.B is a marker of transcriptionally active TSSs.

Previously, we demonstrated that the mouse homolog of H2A.B, designated H2A.B.3, plays a crucial role in regulating transcription and pre-mRNA splicing in a developmentally specific manner in the testis (*5*, *21*, *22*, *24*). Through integrated genomic and proteomic analyses, we proposed a model in which H2A.B.3, localized at intron-exon boundaries, recruits RNA-processing factors to facilitate efficient pre-mRNA splicing (*18*, *21*). In this study, we provide further evidence supporting this model by using N-terminal peptide pull-down assays of H2A.B coupled with SILAC and LC-MS/MS. Specifically, our findings reveal that the unmodified N terminus of H2A.B directly interacts with splicing factors, thereby supporting its proposed role in splicing regulation.

In a more recent study, we demonstrated that H2A.B is enriched in rDNA CHR and plays a critical role in sustaining high levels of RNA Pol I transcription in L1236 and L428 HL cell lines (*8*). Furthermore, H2A.B was essential for maintaining elevated expression levels of ribosomal protein genes (*8*). Through H2A.B co-IP and BioID assays, we identified a direct interaction between H2A.B and the Pol I subunit POLR1A/RPA194 (*8*). These previous findings not only validate the robustness of the SILAC and LC-MS/MS methodology used in the present study—given that Pol I subunits and transcription factors were also detected—but also reveal that these protein-protein interactions might be enhanced by the phosphorylation of H2A.B.

We hypothesize that phosphorylated H2A.B may also interact with RNA Pol II and/or its associated factors. However, given that RNA Pol I transcription is estimated to contribute up to 50% of total RNA synthesis, with even higher levels observed in cancer cells, the relative abundance of RNA Pol I is likely to be greater than that of RNA Pol II. This disparity could limit the detection of Pol II (and/or associated machinery) in our LC-MS/MS experiments. Support for this proposal is that phosphorylated H2A.B is found at RNA Pol II promoters. In addition, in our previous studies using a CHR immunoprecipitation (ChIP)–mass spectrometry approach, we demonstrated an interaction between H2A.B.3 and RNA Pol II in mouse testis nuclei (*21*). Findings from this previous study also revealed that H2A.B-containing nucleosomes may contain the histone variant H3.3 (*21*). This observation may explain the detection of HIRA complex components (HIRA, CABIN1, and UBN1) in our N-terminal phosphopeptide pull-down assays, as this complex is critically involved in the replication-independent deposition of H3.3 into CHR.

One interesting protein to note that arose from our SILAC quantitative proteomics approach of phosphorylation-dependent interacting proteins is proline-rich protein 11 (PRR11). PRR11 is a cell cycle–associated oncogene that has been functionally implicated in the initiation and progression of various malignancies. It exerts oncogenic effects by promoting cellular proliferation (*39*), facilitating epithelial-mesenchymal transition, and enhancing metastasis (*40*). Therefore, investigating the potential involvement of PRR11 in HL may represent a promising focus for future research.

H2A.B does not appear to remain associated with CHR during mitosis. This observation is consistent with the widespread transcriptional repression that characterizes mitotic entry (*37*), as well as the structural reorganization of CHR into a highly compacted state beginning in the prophase, which would be incompatible with H2A.B retention (*22*, *23*). A similar pattern is observed during mouse spermiogenesis: At the developmental stage when transcription is silenced, H2A.B.3 is actively exported from the nucleus, further supporting the notion that H2A.B is excluded from CHR during global repressive states (*21*).

A distinctive feature of histone variants H2A.B and H2A.B.3 is their substantial presence in the nucleoplasm, unbound to CHR. This observation suggests potential non–CHR-specific roles for these variants. Supporting this hypothesis, the N-terminal domain of H2A.B appears to inhibit its full incorporation into CHR, potentially facilitating these additional functions (Fig. 7H). This raises the possibility that the localization and function of nucleoplasmic H2A.B may be modulated by interactions with specific protein or RNA partners under various physiological and pathological conditions. Another nonexclusive explanation could involve the existence of a chaperone that regulates the incorporation of H2A.B into CHR. Furthermore, analyses of H2A.BR7me2a indicate no difference in CHR association compared to the unmodified form, nor do arginine-to-lysine mutations alter its dynamics, suggesting that arginine methylation does not influence the association of H2A.B with CHR (Fig. 7H). Conversely, H2A.BS9ph is exclusively CHR-bound, implying that phosphorylation enhances its CHR stabilization (Fig. 7H). Plausible mechanisms for this stabilization are the inhibition of interactions with factors that otherwise retain H2A.B in the nucleoplasm and/or stabilizing interactions with CHR-associated factors, e.g., RNA Pol I.

In addition to differences in CHR incorporation dynamics, our data reveal that arginine methylation and serine phosphorylation impart distinct functional roles to H2A.B. Notably, H2A.BS9ph is predominantly localized at transcriptionally active TSSs, whereas H2A.BR7me2a is not. Our CUT&RUN analysis identified fewer genomic peaks for H2A.BR7me2a compared to bulk H2A.B and H2A.BS9ph (and being absent from active TSSs). While this result may partially stem from differences in antibody specificity, we propose that H2A.BR7me2a primarily functions not on the genome but within the nucleoplasm, where it likely regulates protein-protein or protein-RNA interactions. Supporting this hypothesis, N-terminal R7me2a peptide pull-down coupled with LC-MS/MS analysis identified preferential interactions between H2A.BR7me2a and subunits of hSWI/SNF compared to unmodified and S9ph peptides in a complex in vitro protein milieu.

Intriguingly, our mass spectrometry analysis also suggests the possibility of a mutually exclusive relationship between H2A.BR7me2a and H2A.BS9ph, as both modifications were never detected simultaneously on the same peptide. This observation suggests a potential cross-talk or regulatory interplay between these two PTMs. However, despite these original observations, the precise functional role of H2A.BR7me2a remains unresolved. Our findings indicate that this modification is not directly recognized by hSWI/SNF, as further discussed below. Equivalently conserved methylated arginine residues are also present in human H2A at R3 and R11, which undergo dimethylation and monomethylation, respectively. This conservation suggests that these methylation sites were preserved throughout the evolutionary divergence of H2A.B. Notably, H2A R3, unlike H2A.B R7, can be both asymmetrically and symmetrically dimethylated (*31*). However, the functional significance of these arginine methylation sites in H2A remains poorly understood.

Our study identifies PRMT1, the predominant enzyme responsible for asymmetric arginine dimethylation in mammalian cells, as the catalyst for H2A.BR7me2a. Furthermore, at least seven distinct isoforms of PRMT1 have been identified, suggesting that each isoform may exhibit unique substrate specificities (*30*, *41*, *42*). In this study, we suggest that this may be the case for H2A.B but cannot rule out the possibility that the responsible PRMT1 is the major isoform that is posttranslationally modified in a specific manner (*43*). In addition, PRMT1, along with PRMT6, mediates asymmetric dimethylation at H2AR3. Despite an exhaustive effort, the kinase(s) responsible for H2A.BS9 phosphorylation remains unidentified, potentially due to functional redundancy among kinases. Ongoing studies aim to address this possibility and elucidate the regulatory landscape of these H2A.B modifications.

A key and unexpected finding of this study is the direct physical and functional interaction between H2A.B and the hSWI/SNF CHR remodeling complex occurring independently of CHR incorporation. This finding reveals a previously uncharacterized epigenetic mechanism in which a histone variant is selectively excluded from CHR to modulate a fundamental process controlling genome architecture—the activity of hSWI/SNF—within a cancer-specific context. These results highlight an additional layer of epigenetic regulation with potential implications for CHR dynamics and transcriptional control in HL.

This conclusion is supported by multiple lines of evidence: (i) N-terminal R7me2a peptide pull-down experiments combined with quantitative proteomics, which identified components of hSWI/SNF; (ii) co-IP assays using CN fractions, in conjunction with proximity-dependent BioID assays, confirming the interaction of H2A.B with hSWI/SNF in a cellular context; (iii) direct binding studies using semiquantitative assays with unmodified and posttranslationally modified H2A.B N-terminal peptides and purified BAFs and PBAFs, which revealed specific interactions; (iv) size exclusion chromatography of CN fractions, indicating the potential coexistence of H2A.B and hSWI/SNF within the same complex; and (v) a marked increase in hSWI/SNF genomic occupancy upon H2A.B knockdown, suggesting a regulatory role for H2A.B in hSWI/SNF recruitment to CHR. These findings collectively suggest that H2A.B functions as a chaperone-like modulator of hSWI/SNF, influencing its genomic deposition. Future cross-linking experiments, coupled with structural studies, will be essential to precisely map the interaction sites between H2A.B and hSWI/SNF.

Our SILAC-based quantitative proteomic analysis revealed that the N-terminal R7me2a peptide preferentially interacts with subunits of the hSWI/SNF complex. This finding raised the intriguing possibility that a shared subunit of the CBAFs and PBAFs could act as a reader protein for this PTM. Supporting this notion is the finding that BRG1 recognizes the H4R3me2a mark, which is critical for the activation of genes that promote colorectal cancer progression (*44*, *45*). However, while CBAF and PBAF display in vitro binding to the R7me2a-modified peptide, their binding affinity is reduced compared to the unmodified peptide. This observation was reproduced when PRMT1 expression was inhibited in HL cells. Perhaps, these observations highlight the dual nature of arginine methylation as an adaptable PTM capable of both facilitating and inhibiting protein-protein interactions. It is possible that R7me2a generally impedes protein-protein interactions, but its inhibitory effects may vary, indirectly shaping the H2A.B interactome by selectively modulating interaction dynamics, e.g., this modification may impede the binding of hSWI/SNF to a lesser extent than other H2A.B-interacting proteins. Not mutually exclusive, the PTM status of interacting proteins may also determine H2A.B partner selectivity.

What is the biological significance of the interaction between hSWI/SNF and H2A.B? One possibility is that this represents an aberrant, cancer-related protein-protein interaction in which H2A.B competes with a physiological regulator of hSWI/SNF function, thereby disrupting normal CHR remodeling. Alternatively, this interaction may play an important role in proper physiological contexts, i.e., in the testis or brain where H2A.B is normally expressed. In conclusion, the N terminus of H2A.B functions as a dynamic protein interaction hub, modulated by its PTM status. In the specific context of HL, the ability of H2A.B to modulate hSWI/SNF activity expands our understanding of its role in CHR dynamics and highlights its important impact on the pathogenesis of this malignancy. Future studies are needed to determine the context-dependent roles of this interaction and its implications for both normal physiology and cancer biology.

## MATERIALS AND METHODS

### Cell culture

L1236 and L428 cells were cultured in RPMI 1640 growth medium supplemented with 10% heat-inactivated fetal bovine serum, 2 mM l-glutamine, and 1% penicillin-streptomycin. Human embryonic kidney 293T cells were cultured in Dulbecco’s modified Eagle’s medium with the same supplements. All cell cultures were incubated at 37°C in a humidified incubator with 5% CO_2_.

### Liquid chromatography–tandem mass spectrometry

For detecting H2A.B PTMs, H2A.B protein was immunoprecipitated from L1236 and L428 cells. IP was performed as previously described (*8*), with minor modifications. Cells (2 × 10^7^) of each type were used per IP. Dynabeads protein A and protein G (80 μl of each) (Thermo Fisher Scientific) were prebound with 8 μg of H2A.B antibody and used for each IP. The immunoprecipitated samples were separated on a precast 4 to 12% bis-tris protein gel and stained with InstantBlue Coomassie protein stain (Expedon) for 2 hour at room temperature. The H2A.B protein band was excised using sterile scalpel blades, followed by destaining, reduction, and alkylation according to standard procedures (*46*). In-gel tryptic digestion and peptide extraction were carried out as previously described (*47*). Peptide extraction solutions were dried in a SpeedVac (Thermo Fisher Scientific) and reconstituted in 20 μl of 0.1% (v/v) formic acid.

The proteolytic peptide samples were subjected to LC-MS/MS analysis on either an LTQ Orbitrap Velos Pro ETD (Thermo Fisher Scientific) or an Orbitrap Fusion Lumos Tribrid (Thermo Fisher Scientific), each interfaced with an UltiMate 3000 HPLC and autosampler system (Dionex). Peptides were separated by nanoscale liquid chromatography, and eluting peptides were ionized using positive ion mode nano–electrospray ionization (ESI) following experimental procedures described previously (*48*). For LTQ Orbitrap Velos Pro ETD experiments, survey scans at a mass/charge ratio (*m*/*z*) 350 to 1750 were acquired in the Orbitrap (resolution of 30,000 at *m*/*z* 400) with an initial accumulation target value of 1 × 10^6^ ions in the linear ion trap; lock mass was applied to polycyclodimethylsiloxane background ions of exact *m*/*z* 445.1200 and 429.0887. The instrument was set to operate in data-dependent acquisition (DDA) mode, and up to the 10 most abundant ions (>5000 counts) with charge states of >+2 were sequentially isolated and fragmented via electron transfer dissociation (ETD) using parameters described previously (*49*). Dynamic exclusion was enabled (exclusion duration, 30 s). For Orbitrap Fusion Lumos Tribrid experiments, survey scans at *m*/*z* 350 to 1500 were recorded in the Orbitrap (resolution of 60,000 at *m*/*z* 200). Peptide ions (>5.0 × 10^3^ counts, charge states of +2 to +6) were sequentially selected for MS/MS via DDA, with the total number of dependent scans maximized within 2-s cycle times. Product ions were generated via ETD and mass analysis in the linear ion trap using the following parameters: ETD reagent time, 100 ms; ETD reagent target, 3.5 × 10^5^; ETD supplemental activation enabled using ETciD with a collision energy of 10%. Dynamic exclusion was enabled and set to the following: *n* times, 1; exclusion duration, 30 s; ±10 parts per million (ppm).

Sequence database searches were performed using the Proteome Discoverer mass informatics platform (version 1.4, Thermo Fisher Scientific) using the search program Mascot (version 2.3, Matrix Science). Peak lists derived from LC-MS/MS were searched using the following parameters: The instrument type was ETD-TRAP; precursor ion and peptide fragment mass tolerances were ±5 ppm and ±0.4 Da respectively; variable modifications included in each search were carbamidomethyl (C), oxidation (M), methyl (R), and dimethyl (R); additional variable modifications included in separate searches were phospho (STY); enzyme specificity was trypsin with up to three missed cleavages; and the Swiss-Prot database (September 2016 release, 552,259 sequence entries) was searched using human sequences only. Peptides determined to be statistically significant according to the Mascot expect metric (*P* < 0.05) were collated. To reduce the incidence of false-positive methylarginine identifications, spectra putatively derived from arginine-methylated peptides were subjected to manual data analysis and spectra lacking evidence for methylarginine-associated neutral losses were discarded as described (*50*).

### Stable isotope labeling by amino acids in cell culture and histone peptide pull-down assays

The peptide pull-down assay was performed as previously described (*51*). Briefly, three SILAC-labeled media were prepared with different combinations of arginine and lysine stable isotopes, designated as “light,” “medium,” and “heavy.” SILAC RPMI 1640 medium (Thermo Fisher Scientific) lacking lysine and arginine was supplemented with 10% dialyzed heat-inactivated fetal bovine serum, 1% l-glutamine, and 1% penicillin-streptomycin. To complete the SILAC medium, l-arginine (50 μg/ml; R0) and l-lysine monohydrochloride (73 μg/ml; L0) as “light isotopes,” an equal molar amount of ^13^C_6_-l-arginine HCl (R6) and 4,4,5,5-D_4_-l-lysine 2HCl (L4) as “medium isotopes,” or an equal molar amount of ^13^C_6_,^15^N_4_-l-arginine HCl (R10) and ^13^C_6_,^15^N_2_-l-lysine HCl (L8) as “heavy isotopes” was added. Isotope-labeled cells (1 × 10^8^) were prepared for nuclear extract isolation. Cells were resuspended in five pellet volumes (2.5 ml) of ice-cold buffer A1 [20 mM Hepes (pH 7.9), 10 mM KCl, and 1.5 mM MgCl_2_] and incubated on ice for 10 min, followed by centrifugation at 200*g* for 5 min at 4°C. The pellet was resuspended in two pellet volumes of ice-cold buffer A2 [10 mM Hepes (pH 7.9), 10 mM KCl, and 1.5 mM MgCl_2_] supplemented with 0.15% NP-40, protease inhibitor cocktail (Sigma-Aldrich), 1 mM 4-(2-aminoethyl)benzenesulfonyl fluoride hydrochloride (AEBSF), and phosphatase inhibitor cocktail (1 mM β-glycerophosphate, 5 mM sodium fluoride, 0.1 mM sodium orthoranadate, and 1 mM sodium pyrophosphate). The suspension was transferred into a prechilled KIMBLE Dounce homogenizer (type B pestle, Sigma-Aldrich) and homogenized with five strokes on ice. The homogenate was spun down for 15 min at 200*g* at 4°C. The pellet was resuspended in two pellet volumes of buffer C [20 mM Hepes (pH 7.9), 420 mM NaCl, 20% glycerol, 2 mM MgCl_2_, 0.2 mM EDTA, 0.1% NP-40, protease inhibitor cocktail (Sigma-Aldrich), 1 mM AEBSF, phosphatase inhibitor cocktail, and 0.5 mM dithiothreitol (DTT)]. The suspension was incubated at 4°C for 1 hour on a rotator, followed by centrifugation at 17,000*g* for 30 min at 4°C. The nuclear extract was stored at −70°C.

N-terminal H2A.B peptides were synthesized by GenScript Biotech (unmodified H2A.B peptide: PRRRRRRGSSGAGGRGRTCSRT; phosphorylated peptide at S9: PRRRRRRG{pSer}SGAGGRGRTCSRT; monomethylated peptide at R7: PRRRRR{MMA}GSSGAGGRGRTCSRT; asymmetrically dimethylated peptide at R7: PRRRRR{ADMA}GSSGAGGRGRTCSRT; monomethylated peptide at R15: PRRRRRRGSSGAGG{MMA}GRTCSRT). Each peptide was C-terminally biotinylated. MyOne Streptavidin C1 beads (75 μl, Thermo Fisher Scientific) were saturated with a 500-μl peptide solution (12.5 μg/ml) and washed three times in 1 ml of protein binding buffer [50 mM tris-HCl (pH 8.0), 150 mM NaCl, 1% NP-40, 0.5 mM DTT, 1× protease inhibitor cocktail (Sigma-Aldrich), 1 mM AEBSF, and phosphatase inhibitor cocktail]. For phosphorylated H2A.B peptide pull-down, the NP-40 concentration was reduced to 0.1%. Beads were resuspended in 800 μl of nuclear extract (0.6 mg/ml) and incubated for 2 hours at 4°C with rotation. Beads were then washed five times with 1 ml of ice-cold binding buffer supplemented with 350 mM NaCl. For combined pull-down experiments (see table S1), beads were pooled and resuspended in 1 ml of ice-cold binding buffer. Proteins were eluted with 40 μl of sample loading LDS buffer (Thermo Fisher Scientific) by incubating at 95°C for 5 min on a thermal mixer at 1200 rpm. Eluted samples were separated on a 4 to 12% bis-tris protein gel, and the entire gel lane was processed for proteolytic peptide preparation.

The proteolytic peptide samples were subjected to LC-MS/MS analysis on a Q Exactive Plus (Thermo Fisher Scientific) interfaced with an UltiMate 3000 HPLC and autosampler system. Peptides were separated by nanoscale liquid chromatography, and eluting peptides were ionized using positive ion mode nano-ESI following experimental procedures described previously (*48*). Survey scans at *m*/*z* 350 to 1750 (automatic gain control target, 1 × 10^6^) were recorded in the Orbitrap (resolution of 70,000 at *m*/*z* 200). The instrument was set to operate in DDA mode, and up to the 12 most abundant ions with charge states of >+2 were sequentially isolated and fragmented via higher-energy collisional dissociation using the following parameters: normalized collision energy, 30; resolution, 17,500; maximum injection time, 125 ms; automatic gain control target, 1 × 10^5^. Dynamic exclusion was enabled (exclusion duration, 30 s).

SILAC data were analyzed using the Proteome Discoverer mass informatics platform, using the search program Mascot for sequence database searches. Peak lists derived from LC-MS/MS were searched using the following parameters: The instrument type was ESI-TRAP; precursor ion and peptide fragment mass tolerances were ±5 ppm and ±0.02 Da, respectively; variable modifications included in each search were carbamidomethyl (C) and oxidation (M); enzyme specificity was trypsin with up to two missed cleavages; and searches were performed against the Swiss-Prot database (July 2017 release, 555,100 sequence entries) using human sequences. Default SILAC 2-plex quantification methods were used to determine heavy (R10, L8-labeled)–to–light (unlabeled), medium (R6, L4-labeled)–to–light, and heavy-to-medium ratios for peptide spectrum matches identified via Mascot. Proteins identified from at least two unique peptides with both log_2_ forward ratio > 1 and log_2_ reverse ratio < −1 were deemed candidate interaction partners. Proteins were also deemed to be candidate interaction partners if they were identified from only modified or only unmodified peptide pull-downs in both “heavy” and “light” samples from least two unique peptides. The biotin-dependent carboxylases were manually discarded because they were known endogenous biotin-binding proteins.

### H2A.B modification–specific antibody generation and specificity testing

The H2A.BR7me2a-, R15me2a-, and S9ph-specific antibodies were generated in rabbit using their respective peptide antigens (GenScript Biotech). The peptide antigens included H2A.BR7me2a: RRR {ADMA}GSSGAGGRC, H2A.BR15me2a: CRGSSGAGG {ADMA} GRT, and H2A.BS9ph: RRRG{pSer}SGAGGRGRTC. Antibody specificity was assessed using a dot-blot assay. Briefly, the modified or unmodified peptides were diluted to 0.025, 0.05, 0.1, 0.25, 0.5, and 2.5 μg/μl, and 2 μl of each was spotted on an Amersham Protran nitrocellulose membrane (GE Healthcare). After air-drying, the membrane was blocked with 3% bovine serum albumin (BSA) for 1 hour. Probing, washing, and detection were performed following the standard Western blot procedures.

### In vitro methylation

In vitro methylation assays of the H2A.B-H2B dimer, prepared as described previously, were performed using a recombinant GST-tagged PRMT1 enzyme purified from bacteria (*52*, *53*). Expression of recombinant GST-PRMT1 fusion protein encoded by pGEX-2TK (*54*) was induced in the *Escherichia coli* strain BL21 with 1 mM isopropyl-β-d-thiogalactopyranoside for 3 hours. Subsequently, bacteria were harvested and lysed in phosphate-buffered saline (PBS) containing 1% Triton X-100 and protease inhibitors (aprotinin and leupeptin; phenylmethylsulfonyl fluoride; Sigma-Aldrich). After sonication, the bacterial supernatant was incubated with glutathione-agarose beads (Machery and Nagel) overnight at 4°C. After washing, bead-bound GST-PRMT1 was eluted [in 50 mM tris-HCl (pH 8) and 25 mM glutathione] and dialyzed (Slide-A-Lyzer Mini Dialysis Units; Thermo Fisher Scientific) against PBS containing 10% glycerol. An eluted GST-tagged PRMT1 enzyme (0.5–1 μg) was incubated with 1 μg of H2A.B-H2B dimer as the substrate in the presence of *S*-adenosyl-methionine (1 mM; New England Biolabs) for 2 hours at 37°C, followed by LC-MS/MS. For radioactive methyltransferase assays, 0.5 to 1 μg of eluted GST-tagged PRMT1 enzyme was incubated with 5 μg of bulk histones (equimolar mixture of the core histones H3, H2A, H2B, and H4) from calf thymus (Sigma-Aldrich) as the positive control or 1 μg of recombinant H2A-H2B, H2A.Z-H2B, or H2A.B-H2B dimer as the substrate in the presence of [^14^C-methyl]-*S*-adenosyl-methionine (20 μCi/ml; PerkinElmer) filled up with PBS to a total volume of 30 μl for 2 hours at 37°C. Subsequently, reactions were separated by SDS–polyacrylamide gel electrophoresis, blotted, and analyzed by autoradiography. Radioactive signals were detected using x-ray films (Hyperfilm; Amersham) and intensifying screens (Kodak). For loading control, membranes were stained with a Ponceau S solution following autoradiography for 10 to 15 min at room temperature and then rinsed with distilled water until the background was destained.

### shRNA-mediated knockdown cell line generation

shRNA-mediated knockdown cell lines were generated via lentiviral transduction, as previously described (*8*). For H2A.B and PRMT6 knockdown, the SMARTvector-inducible shRNA lentiviral vectors (Horizon Discovery) were used: H2A.B shRNA: ATTGAGTACCTGACGGCCA (V3SH11252-229344530) or PRMT6 shRNA: GAGCAAGACACGGACGTTT (V3SH11252-229089275) and nontargeting control shRNA (shNTC; VSC11655). For PRMT1 knockdown, the MISSION PRMT1 shRNA construct (Sigma-Aldrich, TRCN0000290478) in a pLKO.1 vector was used, with the MISSION shNTC construct (Sigma-Aldrich, SHC002) as the nontargeting control.

### H2A.B proximity-dependent BioID cellular assays

The cloning of H2A.B BioID constructs, generation of stable expression cell lines, and BioID assays were performed as previously described (*8*).

### Antibodies and Western blot analysis

Protein samples were supplemented with 10% 2-mercaptoethanol and then heated to 95°C for 10 min. Proteins were separated on a precast Bolt 4 to 12% bis-tris protein gel (Thermo Fisher Scientific) and transferred onto the Immobilon-PSQ 0.2-μm-pore-size polyvinylidene difluoride membrane (Merck Millipore) using the Xcell II wet-transfer module (Thermo Fisher Scientific). Following transfer, the membrane was blocked with 3% BSA in 0.05% PBST (0.05% Tween 20 in 1× PBS). Primary antibody incubation was performed overnight at 4°C in 1% BSA in PBST using the following antibodies: H2A.B, H2A.BR7me2a, H2A.BR15me2a and H2A.BS9ph (custom-made, GenScript Biotech), SDMA (Cell Signaling Technology, 13233S), ADMA (Cell Signaling Technology, 13522S), PRMT1 (Merck Millipore, 07-404), PRMT6 (Cell Signaling Technology, 14641S), SMARCC1 (Cell Signaling Technology, 11956S; Proteintech, 17722-1-A), SMARCE1 (abcam, ab131328), TRA2B (abcam, ab31353), SMARCD1 (abcam, ab224229; Santa Cruz, sc-135843), BRG1 (Santa Cruz, sc-17796x; abcam, ab4081), BRM (Cell Signaling Technology, 11966; abcam, ab15597), ARID1A (Santa Cruz, sc-373784x), ARID2 (Santa Cruz, sc-166117x), DPF2 (Bethyl Laboratories, A303-595A), PHF10 (Thermo Fisher Scientific, PA5-30678; Invitrogen, PA5-30678), β-actin (Cell Signaling Technology, 8457S), and H2A.Z (Active Motif, 39943). After primary antibody incubation, the membrane was washed once with 0.5% PBST containing 0.5 M NaCl for 10 min, followed by a 50-min wash in 0.5% PBST. The membrane was then incubated with secondary antibodies for 1 hour. These included Peroxidase AffiniPure Goat Anti-Rabbit IgG (H + L) (Jackson ImmunoResearch, 111-035-144); Peroxidase IgG Fraction Monoclonal Mouse Anti-Rabbit IgG, light chain specific (Jackson ImmunoResearch, 211-032-171); IRDye 680RD Goat anti-Rabbit IgG secondary antibody (LI-COR Biosciences, 925-68071); IRDye 800CW Donkey anti-Mouse IgG secondary antibody (LI-COR Biosciences, 925-32212); and IRDye 800CW Goat anti-Rabbit IgG secondary antibody (LI-COR Biosciences, 926-32211). Following secondary antibody incubation, the membrane was washed three times in 0.1% PBST. For chemiluminescence detection, the Immobilon Western HRP substrate (Merck Millipore) was applied. Images were acquired using the Amersham 680 imager (GE Healthcare) or the Odyssey CLx Imager (LI-COR Biosciences).

### Cleavage under targets and release using nuclease and data analysis

H2A.BR7me2a and H2A.BS9ph CUT&RUN experiments were performed in L1236 and L428 cells following previously described protocols (*8*). A Rabbit anti-Sheep IgG antibody (Thermo Fisher Scientific, 31240) was used as a control. Sequencing libraries were prepared using the NEBNext Ultra DNA Library Prep Kit for Illumina (New England Biolabs) following the manufacturer’s instructions. The pooled libraries were sequenced with 75 cycles of paired-end reads using the NextSeq 500 sequencer (Illumina). The H2A.BR7me2a and H2A.BS9ph CUT&RUN data were analyzed as previously described (*8*).

### ChIP and data analysis

The BRG1 ChIP assay and library preparation were performed as previously described (*21*). Each ChIP assay was conducted using 5 μl of BRG1 antibody (abcam, ab110641) and 40 μg of CHR. Two replicates of ChIP and input samples were used for library preparation with the NEBNext Ultra DNA Library Prep Kit for Illumina (New England Biolabs) following the kit manual. The resulting libraries were sequenced on an Illumina NextSeq 500 sequencer using paired-end 150-cycle reads. BRG1 ChIP sequencing and input raw data in duplicate were adapter trimmed using Trimmomatic and aligned to the hg38 human genome assembly using Bowtie2 with the default parameters. High-quality, uniquely mapped reads (MAPQ > 20) were deduplicated and used for further analysis. Peak calling was performed using the Genrich peak caller, and differential BRG1 peaks [false discovery rate (FDR) <0.1] between H2A.B knockdown and control were identified using the Diffbind package in Bioconductor.

### Salt-based cellular fractionation and IP

Fractionation was performed as previously described (*21*). Briefly, 1 × 10^7^ cells were lysed in 400 μl of LSBD buffer [50 Hepes (pH 7.0), 3 mM MgCl_2_, 250 mM KCl, 20% glycerol, 1% NP-40, and protease inhibitor cocktail (Sigma-Aldrich)] and homogenized by pipetting. The homogenate was incubated on ice for 30 min, followed by centrifugation at 2500 rpm for 10 min at 4°C. The supernatant was collected as the CN fraction. The pellet was resuspended in 200 μl of LSBD buffer containing 500 mM KCl, homogenized by pipetting, and incubated on ice for 30 min, followed by centrifugation at 2500 rpm for 10 min at 4°C. The supernatant was collected as the loosely bound CHR (LB) fraction. The remaining pellet was resuspended in 200 μl of sample loading LDS buffer (Thermo Fisher Scientific) or 200 μl of LSBD buffer containing 1 M KCl (for subsequent CHR IP experiments) supplemented with 1 μl of Benzonase nuclease (Sigma-Aldrich). The suspension was homogenized by pipetting and incubated at room temperature for 30 min, followed by centrifugation at 17,000*g* for 10 min at 4°C. The supernatant was collected as the CHR fraction. Equal amounts of each fraction were used in Western blot analysis.

For IP experiments, the LB and CHR fractions were diluted with LSBD buffer to a final concentration of 250 mM KCl and supplemented with 1 μl of Benzonase nuclease. Four hundred microliters of the CN, LB, or CHR fraction was used for IPs. Dynabeads protein A and protein G (20 μl each) were prebound with 5 μg of antibodies before use. The antibodies used for IPs included H2A.B, SMARCC1 (Cell Signaling Technology, 19956S), SMARCD1 (Santa Cruz, sc-135843), BRG1 (Cell Signaling Technology, 46360), BRM (Cell Signaling Technology, 11966), ARID1A (Cell Signaling Technology, 12354S), and ARID2 (Cell Signaling Technology, 82342S). Following IP, beads were eluted in 40 μl of sample loading LDS buffer (Thermo Fisher Scientific) by incubating at 80°C for 10 min. Eluted samples were analyzed by Western blot analysis as described previously.

### SWI/SNF complex purification and in vitro peptide binding

Recombinant BAFs were expressed in Gibco Expi293^TM^ cells. Each complex was transiently transfected using Gibco ExpiFectamine Transfection reagent (Thermo Fisher Scientific). Each BAF was transfected with one plasmid containing all corresponding genes with the following exceptions: ARID1A (CBAF), BRD7, BRM, and BRG1 (PBAF), which were cotransfected as appropriate. Each complex was transfected with one plasmid containing all corresponding genes with the following exceptions: ARID1A (CBAF), BRD7, BRM, and BRG1 (PBAF), which were cotransfected as appropriate. Complexes were purified by nuclear extraction and subsequent FLAG bead pull-down. The elution buffer contained 20 mM Hepes (pH 8.0), 50 to 100 mM KCl, 5% glycerol, 0.05% NP-40, 0.5 mM DTT, and 3×FLAG peptide (250 ng/μl). Complexes were concentrated to ~1 to 3 μM before freezing in liquid nitrogen and storage at −80°C. Tested complexes were PBAF with BRG1, PBAF with BRM, CBAF with BRG1, and CBAF with BRM.

For peptide and recombinant nucleosome binding assay, 5 μl of Dynabeads MyOne Streptavidin T1 (Thermo Fisher Scientific) was used per binding assay in a 50-μl volume. G100++ and G350++ buffers contained 20 mM Hepes (pH 8.0), 100 or 350 mM KCl, 2 mM MgCl_2_, 10 μM ZnSO_4_, 5% glycerol, 0.05% NP-40, and, added fresh, BSA (100 μg/ml) and 0.5 mM β-mercaptoethanol. G100++ was used for peptide and BAF binding steps. Threefold molar excess of each peptide in G100++ was incubated with Dynabeads at 4°C for 30 to 45 min with rotation and washed three times with 100 μl of G100++ buffer. BAFs were mixed with the peptide containing beads at 12.5 nM in 50 μl of G100++ and incubated for 90 min in G100++ buffer. Dynabeads with peptide and BAF were washed three times in 100 μl of G350++ to remove unbound BAFs. The Dynabeads were resuspended in 50 μl of 2× Laemmli buffer and heated to 95°C for 5 min. The samples were stored at −20°C. The bound proteins/complexes were separated on 4 to 20% gradient SDS–polyacrylamide gel electrophoresis gels and transferred to nitrocellulose for Western blotting. Western blots were prepared and detected using the LI-COR fluorescence method. Blocking and antibody solutions were in PBST with 1:5 Fish Serum Blocking Buffer (Thermo Fisher Scientific). All wash steps were in PBST. Blots were rinsed and stored in PBS until scanning on LI-COR’s Odyssey CLx. Blots were imaged and quantified using Image Studio Lite version 5.2.

The C-terminal biotinylated peptides used in the assay included H2A N-terminal peptide: SGRGKQGGKARAKAKTRS; H2A.Z N-terminal peptide: AGGKAGKDSGKAKTKAVSRS; phosphorylated S9 H2A.B N-terminal peptide; monomethylated R7 H2A.B N-terminal peptide; asymmetrically dimethylated H2A.B R7 N-terminal peptide at R7; monomethylated R15 H2A.B N-terminal peptide; and unmodified H2A.B N-terminal peptide as used in SILAC-peptide pull-down experiments. In addition, biotinylated mononucleosomes assembled from recombinant human histones (EpiCypher, 16-0006) were tested.

### Gel filtration chromatography

Gel filtration chromatography of the CN fraction isolated from L428 cells was performed using a Superose 6 Increase 10/300 GL column (Cytiva) mounted on an ÄKTA Go protein purification system (Cytiva). The CN fraction (400 μl) was prepared from 1 × 10^7^ cells as described above. The column was equilibrated with the ice-cold elution buffer [50 mM Hepes-NaOH (pH 7.0), 3 mM MgCl_2_, 250 mM KCl, and 10% glycerol]. Before injection, the CN fraction was adjusted to 500 μl by adding LSBD buffer supplemented with 1× protease inhibitor cocktail (Sigma-Aldrich) and 0.5 μl of Pierce universal nuclease (Thermo Fisher Scientific). The sample was loaded onto the column, and the elution was carried out at a constant flow rate of 0.5 ml/min. Fractions of 1 ml were collected. Molecular weight calibration was performed using gel filtration protein standards (Sigma-Aldrich), including blue dextran (2000 kDa), thyroglobulin (670 kDa), β-amylase (200 kDa), and cytochrome c (13 kDa). After collection, each fraction was supplemented with 1× protease inhibitor cocktail (Sigma-Aldrich). Elution fractions were purified by trichloroacetic acid precipitation. Briefly, 100 μl of 0.15% deoxycholate was added to each fraction and incubated at room temperature for 10 min. A 50-μl trichloroacetic acid solution was added to each fraction, followed by 30-min incubation on ice. The protein precipitate was pelleted by centrifugation at 10,000*g* for 10 min at 4°C, followed by washing in 500 μl of ice-cold acetone. The pellet was resuspended in 2× LDS sample buffer and used for Western blot analysis as described above.

### Establishment of stable cell lines expressing H2A.B or mutants

Gene fragments encoding H2A.B or its mutants were in vitro synthesized (Thermo Fisher Scientific) and cloned into the pLVX-EF1α-IRES-Neo lentiviral vector. Lentiviral transduction was performed as previously described (*8*). Transduced L1236 and L428 cells were selected with G-418 (0.5 and 1 mg/ml, respectively; Sigma-Aldrich) for 6 days. The selected cells were subsequently used for cellular fractionation and Western blot analysis.

### Immunofluorescence

Immunofluorescence immunostaining of asynchronously growing L428 cells was performed as previously described (*8*). For G_2_-M phase synchronization, 1 × 10^6^ cells were treated with nocodazole (100 ng/ml) for 24 hours. Cells were then pelleted and resuspended in 1 ml of Dulbecco’s PBS (Sigma-Aldrich). A 150-μl aliquot was applied to each glass slide and centrifuged using a Cytospin at 500 rpm for 5 min. The cells were fixed in 4% paraformaldehyde in PBS (pH 7.4) for 15 min at room temperature. Permeabilization was carried out using 0.25% Triton X-100 in PBS for 10 min, followed by blocking in 3% BSA for 60 min at room temperature. H2A.B and H2A.BS9ph primary antibodies were diluted in blocking buffer and applied to the slides overnight at 4°C. The slides were washed three times for 5 min each in PBS and then incubated with appropriate secondary antibodies for 1 hour at room temperature. The slides were then washed three times in PBS, and nuclei were counterstained with DAPI (4′,6-diamidino-2-phenylindole; 1 μg/ml) in PBS for 1 min at room temperature. The slides were mounted with Vectashield mounting medium and sealed with coverslips. Imaging was performed using a Leica SP5 confocal microscope (Leica Camera).

### RNA-seq data analysis

The RNA sequencing (RNA-seq) data were processed as previously described (*8*). Briefly, the RNA-seq raw data in three replicates were adapter trimmed using Trimmomatic (*55*) and mapped to the *Homo sapiens* (hg38) genome assembly using HISAT2 (*56*). Gene annotation was obtained from the UCSC hg38 gene annotation in iGenomes. The sequencing reads were assigned to genes using the featureCounts function in Rsubread package in Bioconductor (*57*).

### ATAC-seq data analysis

The ATAC-seq data were processed as previously described (*8*). Briefly, the raw data were adapter trimmed using Trimmomatic and mapped to the hg38 human genome assembly using Bowtie2 with the default parameters. High-quality, uniquely mapped reads (MAPQ > 20) were deduplicated using Picard. Peak calling of accessible regions was performed by Genrich peak caller with parameter setting (Genrich -t -o -f -r -j -y -d 100 -q 0.05 -e chrM -v). The peaks were annotated by the HOMER package. ATAC-seq differential accessibility analysis on the ATAC-seq peaks between H2A.B knockdown versus control was performed with the DEseq2 R package.
