# Supplementary material for: Nonchromatin regulatory functions of the histone variant H2A.B in SWI/SNF genomic deposition
Source: Sci Adv. 2025 Jul 25;11(30):eadx1568. doi: 10.1126/sciadv.adx1568 (PMC12292949; doi:10.1126/sciadv.adx1568)
Supplement: Supplementary file 1 — Figs. S1 to S13 Tables S1 to S7 [file sciadv.adx1568_sm.pdf]

Science Advances
AAAS

Supplementary Materials for

Nonchromatin regulatory functions of the histone variant H2A.B in SWI/SNF genomic deposition

Xuanzhao Jiang *et al.*

Corresponding author: Tatiana A. Soboleva, tanya.soboleva@anu.edu.au;
David J. Tremethick, david.tremethick@anu.edu.au

Sci. Adv. **11**, eadx1568 (2025)
DOI: 10.1126/sciadv.adx1568

This PDF file includes:

Figs. S1 to S13
Tables S1 to S7

A

MPRRRRRRGS SGAGGRGRTC SRTVRAELSF SVSQVERSLR EGHYAQRLSR TAPVYLAAVI 1-60
EYLTAKVLEL AGNEAQNSGE RNITPLLLDM VVHNDRLLST LFNTTTISQV APGED 61-115

B

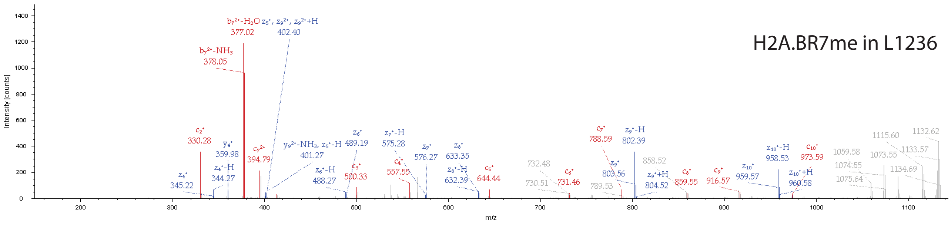

#	c	Seq.	z	#
1	174.1349	R		11
2	330.2361	R	959.5130	10
3	500.3528	R*	803.4118	9
4	557.3743	G	633.2951	8
5	644.4063	S	576.2736	7
6	731.4383	S	489.2416	6
7	788.4598	G	402.2096	5
8	859.4969	A	345.1881	4
9	916.5184	G	274.1510	3
10	973.5398	G	217.1295	2
11		R	160.1081	1

* methylation site

C

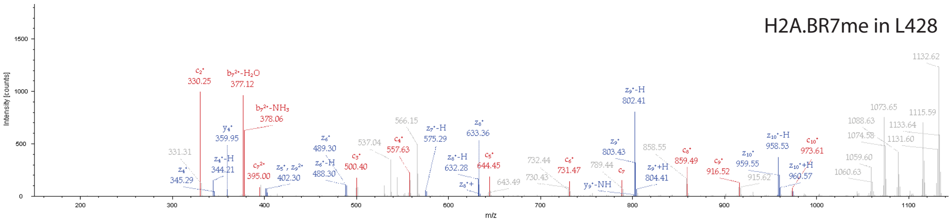

#	c	Seq.	z	#
1	174.1349	R		11
2	330.2361	R	959.5130	10
3	500.3528	R*	803.4118	9
4	557.3743	G	633.2951	8
5	644.4063	S	576.2736	7
6	731.4383	S	489.2416	6
7	788.4598	G	402.2096	5
8	859.4969	A	345.1881	4
9	916.5184	G	274.1510	3
10	973.5398	G	217.1295	2
11		R	160.1081	1

* methylation site

D

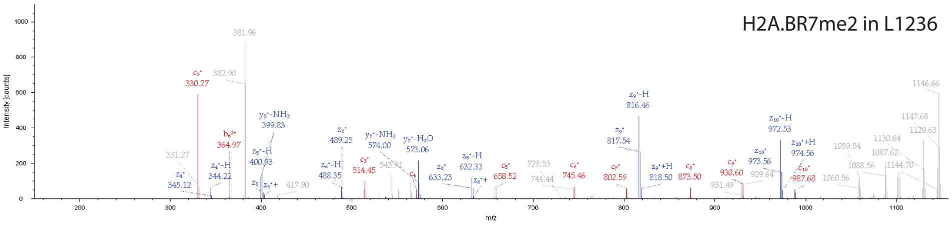

#	c	Seq.	z	#
1	174.1349	R		11
2	330.2361	R	973.5286	10
3	514.3685	R**	817.4275	9
4	571.3899	G	633.2951	8
5	658.4220	S	576.2736	7
6	745.4540	S	489.2416	6
7	802.4754	G	402.2096	5
8	873.5126	A	345.1881	4
9	930.5340	G	274.1510	3
10	987.5554	G	217.1295	2
11		R	160.1081	1

** dimethylation site

E

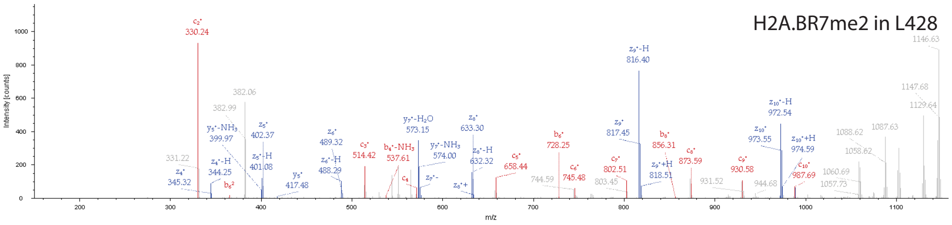

#	c	Seq.	z	#
1	174.1349	R		11
2	330.2361	R	973.5286	10
3	514.3685	R*	817.4275	9
4	571.3899	G	633.2951	8
5	658.4220	S	576.2736	7
6	745.4540	S	489.2416	6
7	802.4754	G	402.2096	5
8	873.5126	A	345.1881	4
9	930.5340	G	274.1510	3
10	987.5554	G	217.1295	2
11		R	160.1081	1

** dimethylation site

F

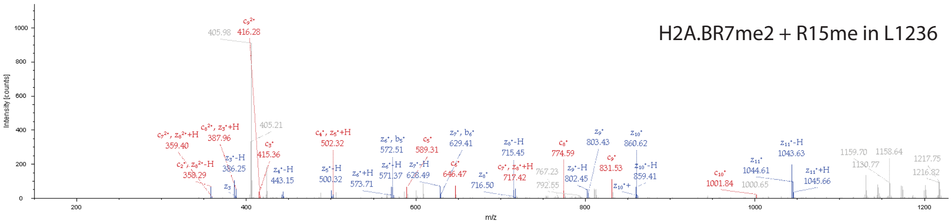

#	c	Seq.	z	#
1	174.1349	R		11
2	358.2674	R**	1044.5657	10
3	415.2888	G	860.4333	9
4	502.3208	S	803.4118	8
5	589.3529	S	716.3798	7
6	646.3743	G	629.3478	6
7	717.4115	A	572.3263	5
8	774.4329	G	501.2892	4
9	831.4544	G	444.2677	3
10	1001.5711	R*	387.2463	2
11	1058.5926	G	217.1295	1
		R	160.1081	

* methylation site ** dimethylation site

G

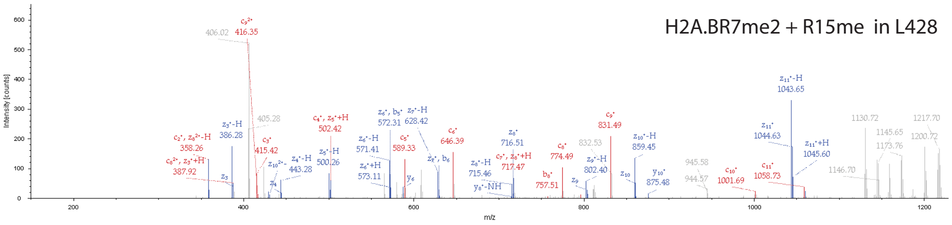

#	c	Seq.	z	#
1	174.1349	R		11
2	358.2674	R**	1044.5657	10
3	415.2888	G	860.4333	9
4	502.3208	S	803.4118	8
5	589.3529	S	716.3798	7
6	646.3743	G	629.3478	6
7	717.4115	A	572.3263	5
8	774.4329	G	501.2892	4
9	831.4544	G	444.2677	3
10	1001.5711	R*	387.2463	2
11	1058.5926	G	217.1295	1
		R	160.1081	

* methylation site ** dimethylation site

Fig. S1. LC-MS/MS uncovers arginine methylation at the N-terminal tail of H2A.B in HL cells. (**A**) The sequence coverage from a LC-MS/MS analysis of immunoprecipitated H2A.B protein from L1236 and L428 cells. Residues covered in L1236 samples are in bold, and those covered in L428 samples are underlined. (**B**-**G**). Electron-transfer dissociation (ETD) tandem MS spectra of H2A.B derived tryptic peptides, observed as $[M + 3H]^{3+}$; RR(monomethyl-R)GSSGAGGR spectra obtained from L1236 cells (B) and L428 cells (C); RR(dimethyl-R)GSSGAGGR spectra obtained from L1236 cells (D) and L428 cells (E); and R(dimethyl-R)GSSGAGG(monomethyl-R)GR spectra obtained from L1236 cells (F) and L428 cells (G). Fragment ions are labelled as follows: c' and b ions (red), z'• and y ions (blue). Prominent ions resulting from $-NH_3$ or $-H_2O$ losses are also labelled. Theoretical masses of c' and z'• ions are shown to the right of the spectrum. The observed c' and z'• ions are in bold and coloured.

A

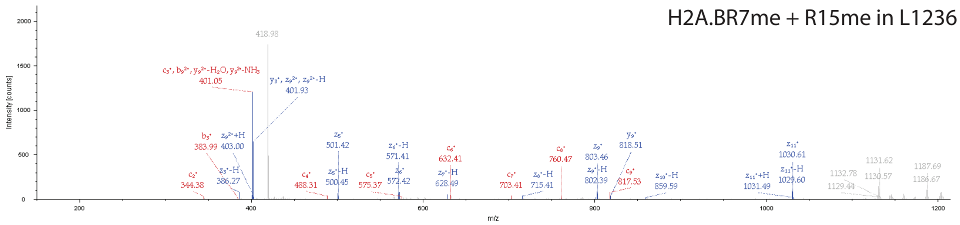

#	c	Seq.	z	#
1	174.1349	R		11
2	344.2517	R*	1030.5501	10
3	401.2732	G	860.4333	9
4	488.3052	S	803.4118	8
5	575.3372	S	716.3798	7
6	632.3587	G	629.3478	6
7	703.3958	A	572.3263	5
8	760.4173	G	501.2892	4
9	817.4387	G	444.2677	3
10	987.5555	R*	387.2463	2
11	1044.5770	G	217.1295	1
		R	160.1081	

* methylation site

B

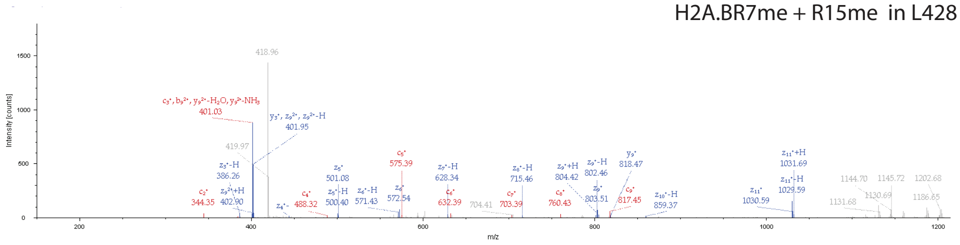

#	c	Seq.	z	#
1	174.1349	R		11
2	344.2517	R*	1030.5501	10
3	401.2732	G	860.4333	9
4	488.3052	S	803.4118	8
5	575.3372	S	716.3798	7
6	632.3587	G	629.3478	6
7	703.3958	A	572.3263	5
8	760.4173	G	501.2892	4
9	817.4387	G	444.2677	3
10	987.5555	R*	387.2463	2
11	1044.5770	G	217.1295	1
		R	160.1081	

* methylation site

C

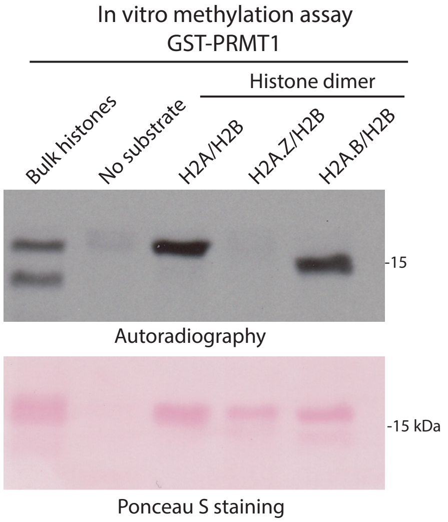

D

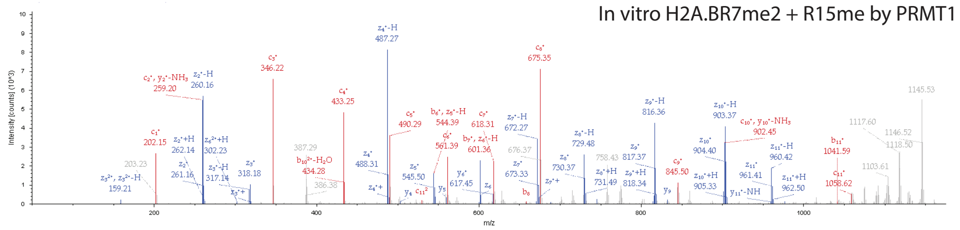

#	c	Seq.	z	#
1	202.1662	R**		12
2	259.1877	G	961.4810	11
3	346.2197	S	904.4595	10
4	433.2518	S	817.4275	9
5	490.2732	G	730.3955	8
6	561.3103	A	673.3740	7
7	618.3318	G	602.3369	6
8	675.3533	G	545.3154	5
9	845.4700	R*	488.2940	4
10	902.4915	G	318.1772	3
11	1058.5926	R	261.1557	2
12		T	105.0546	1

* methylation site ** dimethylation site

Fig. S2. H2A.B is methylated by PRMT1 *in vitro*. (**A-B**) Electron-transfer dissociation (ETD) tandem MS spectra of H2A.B derived tryptic peptide R(monomethyl-R)GSSGAGG(monomethyl-R)GR (observed as $[M + 3H]^{3+}$) obtained from L1236 cells (A) and L428 cells (B). (**C**) GST-tagged PRMT1 was subjected to *in vitro* methyltransferase assays using bulk calf histones, recombinant H2A–H2B, H2A.Z–H2B or H2A.B–H2B dimers as substrates. The methylation reactions were started upon addition of [14C-methyl]-SAM and incubated for 2 hours. As negative control, GST-PRMT1 enzyme and [14C-methyl]-SAM were incubated without substrate (no substrate). Subsequently, samples were separated by SDS-PAGE, blotted and analysed by autoradiography. Ponceau S staining served as loading control. (**D**) ETD tandem MS spectrum of the peptide (dimethyl-R)GSSGAGG(methyl-R)GRT, derived from a WaLP digestion of H2A.B methylated *in vitro* by PRMT1. Fragment ions are labelled as follows: c' and b ions (red), $z^{\prime\bullet}$ and y ions (blue). Prominent ions resulting from $-NH_3$ or $-H_2O$ losses are also labelled. Theoretical masses of c' and $z^{\prime\bullet}$ ions are shown to the right of the spectrum. The observed c' and $z^{\prime\bullet}$ ions are in bold and coloured.

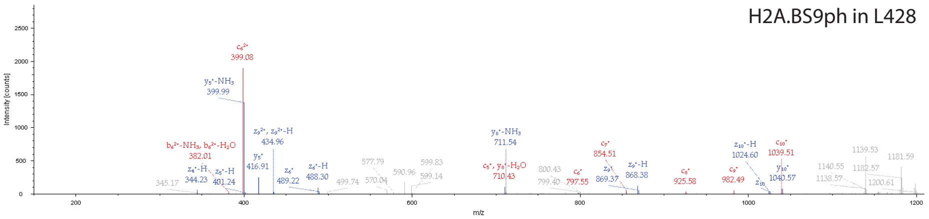

#	c	Seq.	z	#
1	174.1349	R		11
2	330.2361	R	**1025.4636**	10
3	486.3372	R	**869.3625**	9
4	543.3586	G	713.2614	8
5	**710.3570**	S*	656.2400	7
6	**797.3890**	S	**489.2416**	6
7	**854.4105**	G	402.2096	5
8	**925.4476**	A	345.1881	4
9	**982.4691**	G	274.1510	3
10	**1039.4905**	G	217.1295	2
11		R	160.1081	1

* phosphorylation site

Fig. S3. H2A.B is phosphorylated at S9 site in HL cells. The electron-transfer dissociation (ETD) tandem MS spectrum of H2A.B derived tryptic peptide RRRG(phospho-S)SGAGGR (observed as [M + 3H]$^{3+}$) obtained from L428 cells. Fragment ions are labelled as follows: c' and b ions (red), z'• and y ions (blue). Prominent ions resulting from –NH_3 or –H_2O losses are also labelled. Theoretical masses of c' and z'• ions are shown to the right of the spectrum. The observed c' and z'• ions are in bold and coloured.

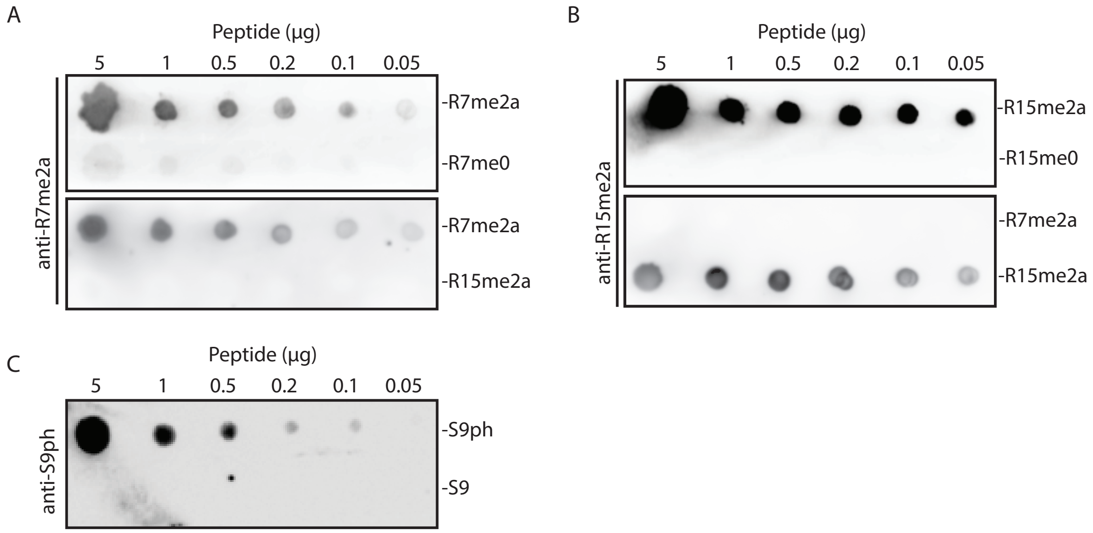

Fig. S4. Development of H2A.B R7me2a and S9ph specific antibodies. (**A-C**) Dot blot analyses demonstrating the specificity of anti-R7me2a (A), control anti-R15me2a (B), and anti-S9ph rabbit antibodies (C). In each panel, 0.05-5 µg of each modified and unmodified peptides were spotted onto a nitrocellulose membrane in parallel. The membranes were probed with the respective antibodies to assess their specificity.

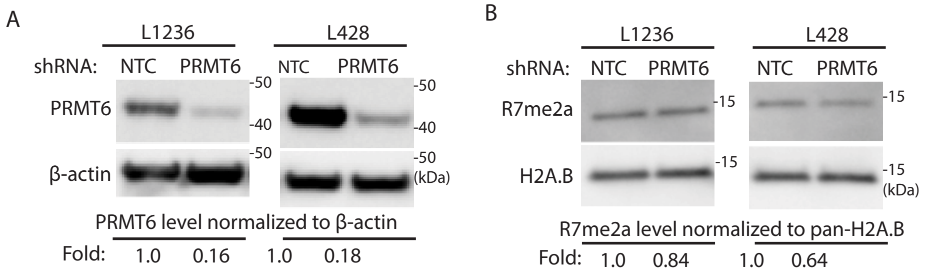

Fig. S5. PRMT6 is not the major arginine methyltransferase responsible for H2A.B R7 asymmetric di-methylation. (**A**) A Western blot analysis of PRMT6 levels in non-targeting control (NTC) or PRMT6 shRNA-transduced L1236 cells (left panel) and L428 cells (right panel). β-actin serves as a loading control. (**B**) A Western blot analysis of R7me2a levels in H2A.B immunoprecipitates from NTC or PRMT6 shRNA-transduced L1236 cells (left panel) and L428 cells (right panel, N=2).

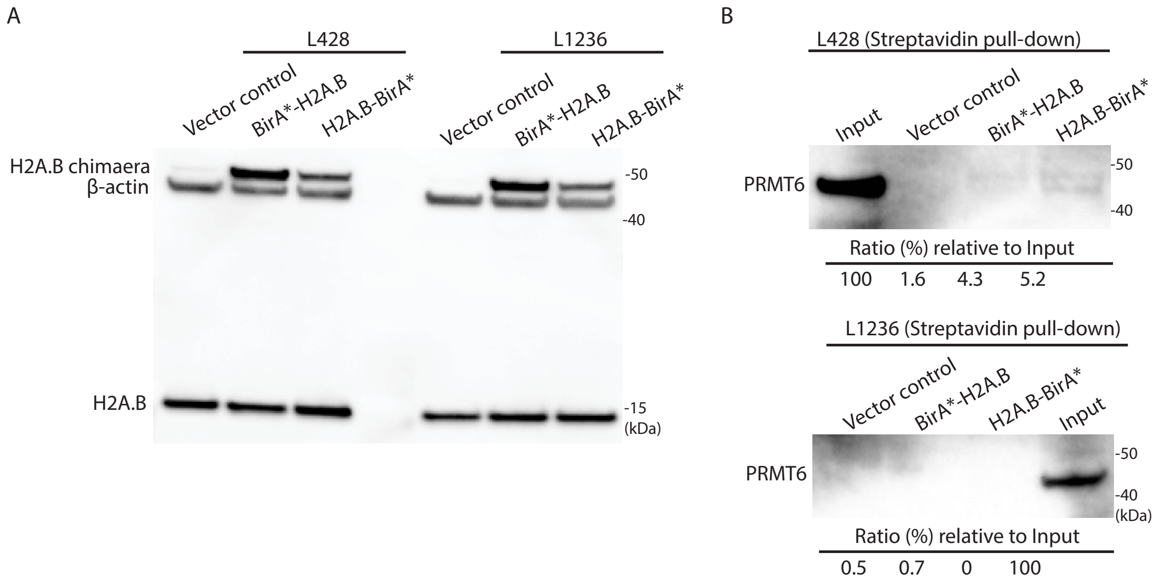

Fig. S6. PRMT6 does not interact with H2A.B in cells. (**A**) A Western blot confirming the expression of BioID constructs, BirA*-H2A.B and H2A.B-BirA*, in L428 and L1236 cells using anti-H2A.B antibody. β-actin serves as a loading control. (**B**) Western blot detection of PRMT6 from streptavidin affinity purifications from biotin-labelled L428 (left panel) and L1236 (right panel) cell lysates. The positive control was loaded as 1% (v/v) input.

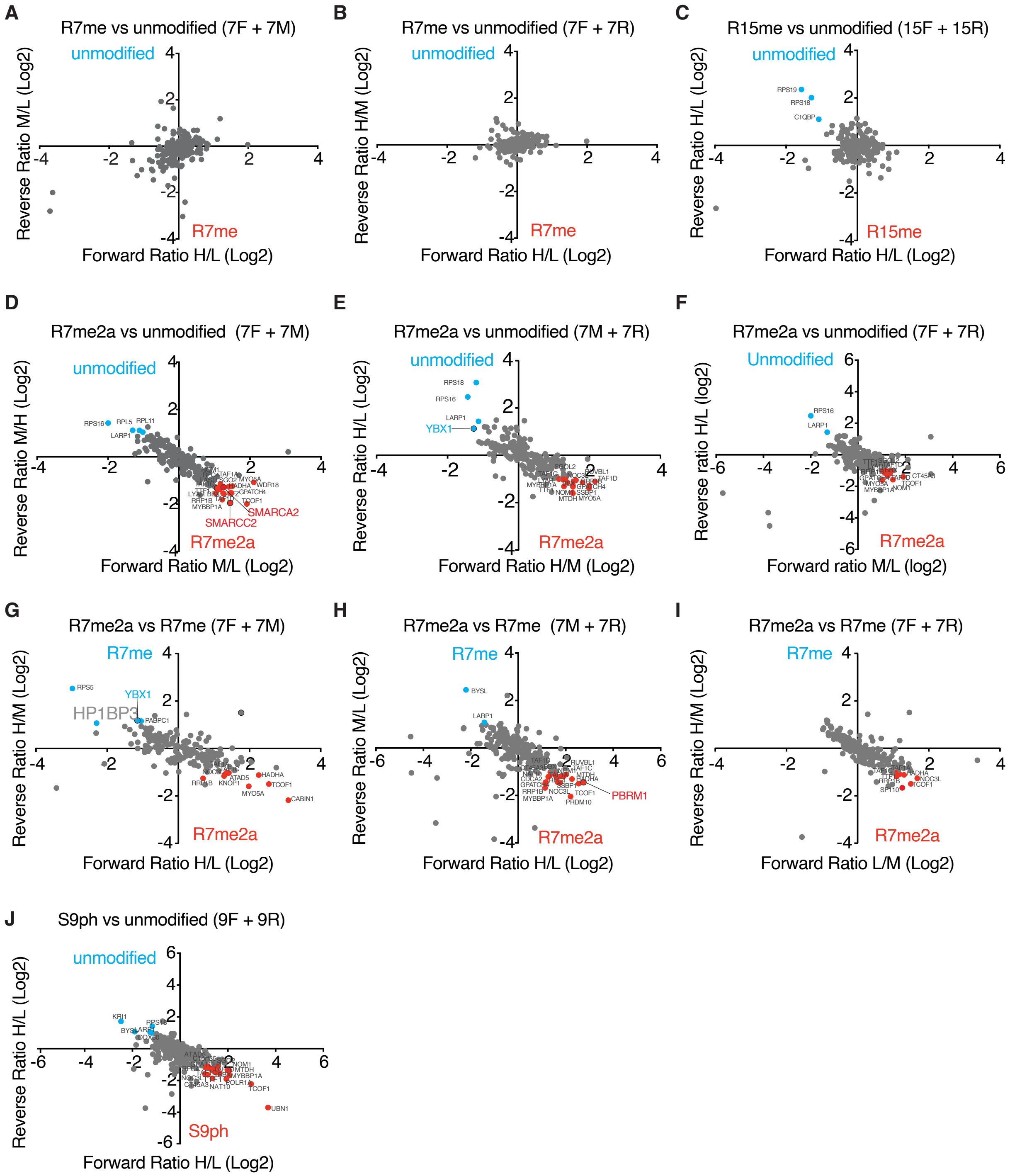

A
R7me vs unmodified (7F + 7M)
unmodified
R7me
Reverse Ratio M/L (Log2)
Forward Ratio H/L (Log2)
B
R7me vs unmodified (7F + 7R)
unmodified
R7me
Reverse Ratio H/M (Log2)
Forward Ratio H/L (Log2)
C
R15me vs unmodified (15F + 15R)
unmodified
R15me
Reverse Ratio H/L (Log2)
Forward Ratio H/L (Log2)
D
R7me2a vs unmodified (7F + 7M)
unmodified
SMARCA2
SMARCC2
R7me2a
Reverse Ratio M/H (Log2)
Forward Ratio M/L (Log2)
E
R7me2a vs unmodified (7M + 7R)
unmodified
YBX1
R7me2a
Reverse Ratio H/L (Log2)
Forward Ratio H/M (Log2)
F
R7me2a vs unmodified (7F + 7R)
Unmodified
R7me2a
Reverse ratio H/L (log2)
Forward ratio M/L (log2)
G
R7me2a vs R7me (7F + 7M)
R7me
YBX1
HP1BP3
R7me2a
Reverse Ratio H/M (Log2)
Forward Ratio H/L (Log2)
H
R7me2a vs R7me (7M + 7R)
R7me
PBRM1
R7me2a
Reverse Ratio M/L (Log2)
Forward Ratio H/L (Log2)
I
R7me2a vs R7me (7F + 7R)
R7me
R7me2a
Reverse Ratio H/M (Log2)
Forward Ratio L/M (Log2)
J
S9ph vs unmodified (9F + 9R)
unmodified
S9ph
Reverse Ratio H/L (Log2)
Forward Ratio H/L (Log2)

Fig. S7. Post-translational modifications reshape the interactome of H2A.B. (A-J) Scatter plots depicting the Log2 forward and reverse ratios of proteins quantified from a SILAC-based peptide pull-down assay. Cells were labelled with three sets of stable arginine and lysine isotopes, "Light" (L): Arg-0 and Lys-0; "Medium" (M): Arg-6 and Lys-4); and "Heavy" (H): Arg-10 and Lys-8. Forward and reverse ratios were calculated from pairs of isotope labels (H/L, H/M or M/L) in the samples as detailed in Table S1. Each plot compares the two ratios for specific modifications. This includes R7me vs unmodified ratios quantified from samples 7F and 7M (A) and 7F and 7R (B); R15me vs unmodified ratios from samples 15F and 15R (C); R7me2a vs unmodified ratios from samples 7F and 7M (D), 7M and 7R (E) and 7F and 7R (F); R7me2a vs R7me ratios from samples 7F and 7M (G), 7M and 7R (H) and 7F and 7R (I). S9ph vs unmodified ratios were quantified from samples 9F and 9R (J). Highlighted are the significant protein readers of the modified peptide with Log2 forward ratio > 1 and Log2 reverse ratio < -1 (red dots), and significant readers of the unmodified peptide with Log2 forward ratio < -1 and Log2 reverse ratio > 1 (blue dots).

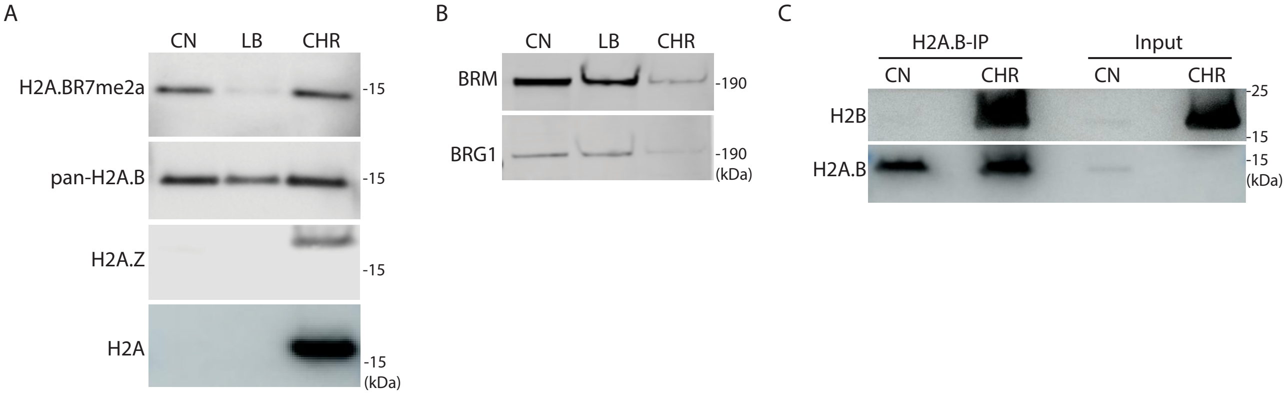

Fig. S8. H2A.BR7me2a and hSWI/SNF are present in a non-chromatin-bound state. (**A**) A Western blot analysis of the subcellular location of H2A.B and H2A.BR7me2a. H2A.B was immunoprecipitated from L428 cytoplasmic/nucleoplasmic (CN), loosely bound to chromatin (LB), and chromatin (CHR) fractions. The Western blot was then probed with anti-H2A.B, anti-H2A.BR7me2a, anti-H2A.Z or anti-H2A antibodies**.** (**B**) Western blot detection of hSWI/SNF core catalytic subunits BRM and BRG1 across the different L428 subcellular fractions. (**C**) A Western blot analysis of H2B following the immunoprecipitation of H2A.B from the CN and CHR fraction.

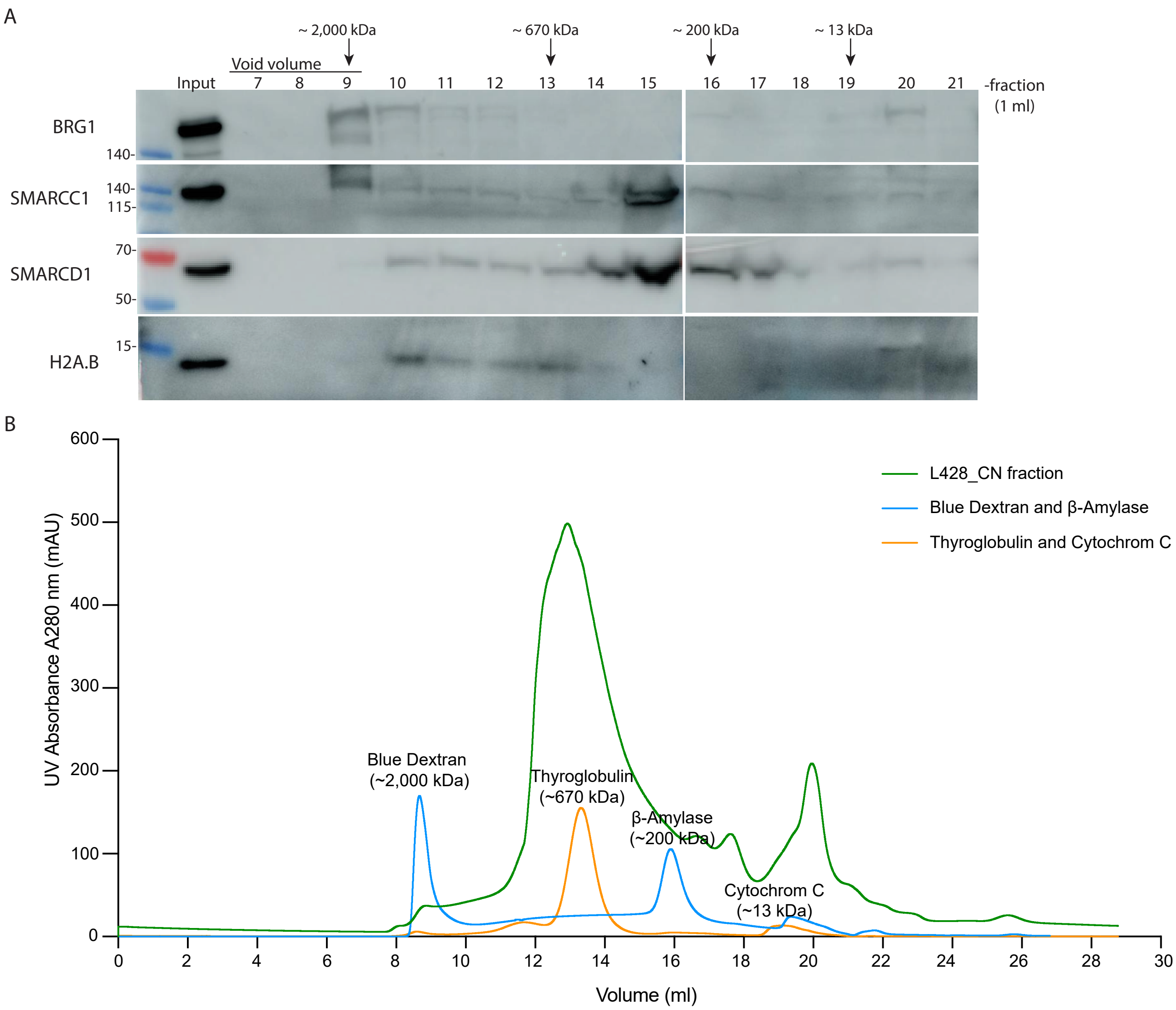

Fig. S9. H2A.B co-fractionates with hSWI/SNF. (**A**) Western blot analyses of H2A.B, BRG1, SMARCC1, and SMARCD1 in the cytoplasmic/nucleoplasmic fractions of L428 cells following gel filtration chromatography. Fractions 7 to 21, covering the major absorbance peaks were analysed. We note that a significant proportion of SMARCC1 and SMARCD1 are not assembled into the fully assembled hSWI/SNF complex indicating the existence of subcomplexes. Interestingly, H2A.B does not associate with these subcomplexes. (**B**) Gel filtration chromatogram of L428 cytoplasmic/nucleoplasmic fraction combined with the elution profile of molecular weight standards: Blue Dextran (~2,000 kDa), Thyroglobulin (~670 kDa), β-Amylase (~200 kDa), and Cytochrom C (~13 kDa).

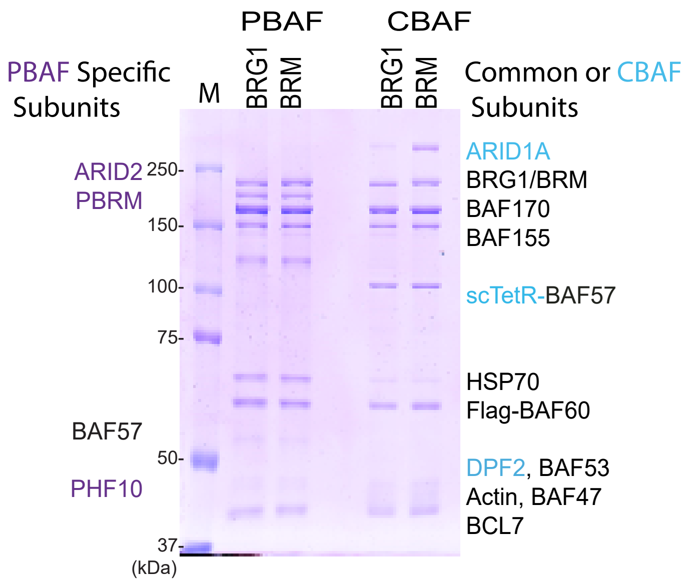

Fig. S10. Purified PBAF and CBAF complexes. PBAF (BRG1 and BRM) or CBAF (BRG1 or BRM) subunit expression constructs were transfected into GibcoTM Expi293TM cells. Complexes were purified by Flag-tagged protein purification.

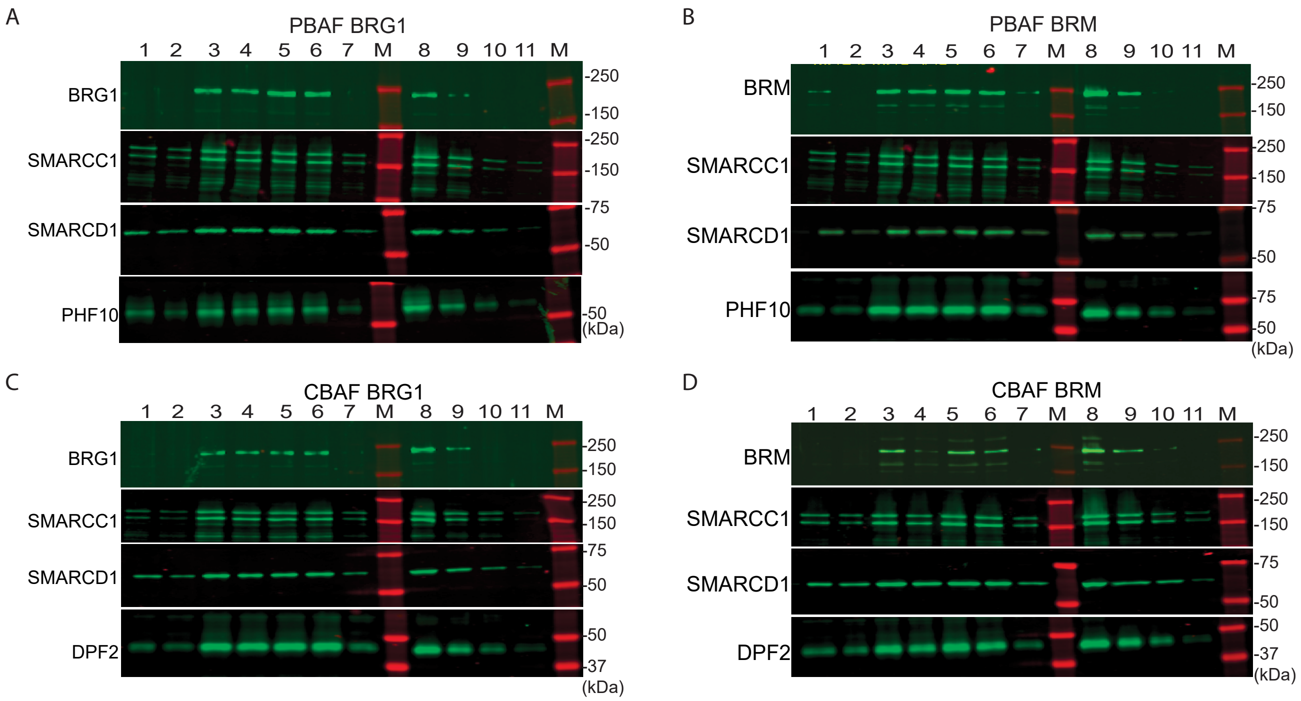

Fig. S11. A Western blot analysis of the binding of PBAF and CBAF to unmodified and arginine methylated H2A.B N-terminal peptides. H2A, H2A.Z, H2A.B, H2A.BR7me2a, H2A.BR7me or H2A.BR15me biotin tagged N-terminal peptides were incubated with purified recombinant PBAF (BRG1, panel A; BRM, panel B) or CBAF (BRG1, panel C; BRM, panel D). Peptide-bound hSWI/SNF complexes were subjected to a Western blot analysis and relative peptide binding affinities were determined using common (BRG1, BRM, SMARCC1 and SMARCD1) and subunit specific CBAF (DPF2) and PBAF (PHF10) antibodies. The relative CBAF or PBAF binding affinity to a biotin tagged peptide was quantified by a comparison of the binding affinity of each individual antibody with a two-fold dilution series of input complex (50%-6.25%). Lane 1, H2A; Lane 2, H2A.Z; lane 3, H2A.B; lane 4, H2A.BR7me2a; Lane 5, H2A.BR7me; Lane 6, H2A.BR15me; Lane 7, human nucleosome cores; Lane 8, 50% input; Lane 9, 25% input; Lane 10, 12.5% input; Lane 10, Lane 11, 6.25% input. M, molecular weight markers.

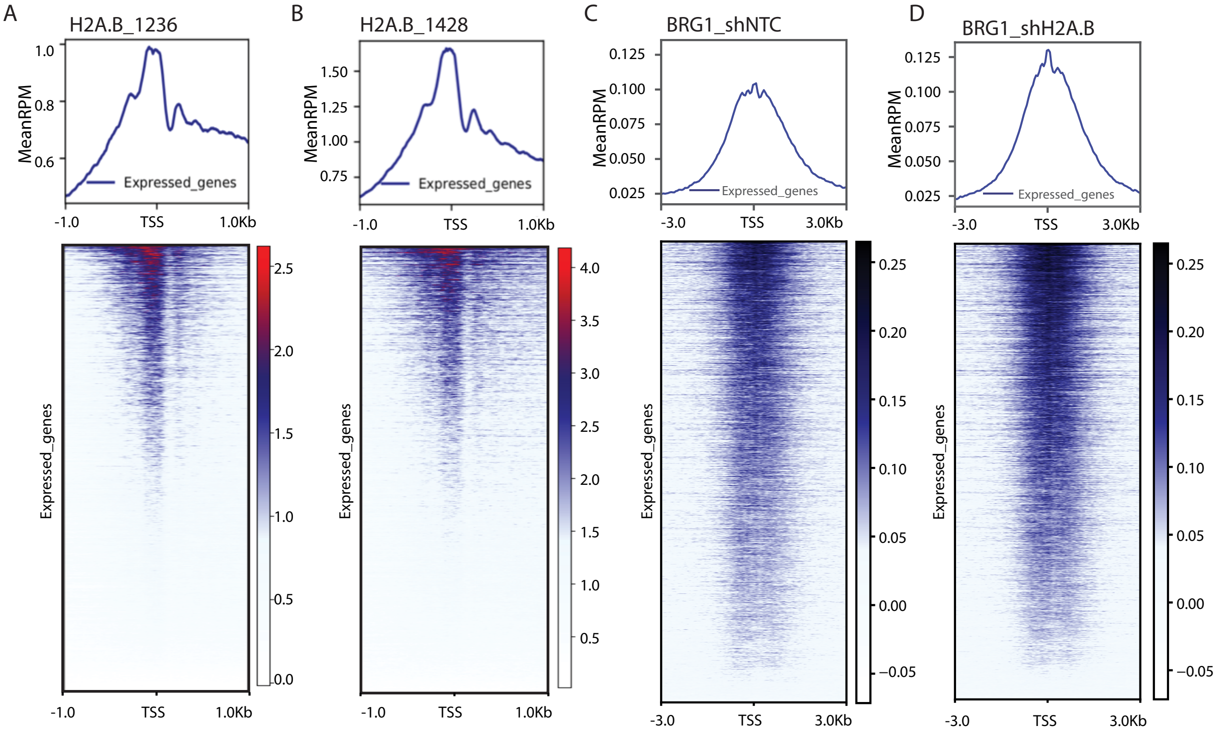

Fig. S12. H2A.B and BRG1 are targeted to the TSSs of active genes. (**A**) A meta-gene plot showing H2A.B mean CUT&RUN coverage aligned between -1 and +1 kb from the TSS of expressed genes (Log2 TPM > 3) in L1236 cells. (**B**) A meta-gene plot showing H2A.B mean CUT&RUN coverage aligned between -1 and +1 kb from the TSS of expressed genes (Log2 TPM > 3) in L428 cells. (**C**) A meta-gene plot showing mean BRG1 ChIP-seq coverage aligned between -3 kb to 3 kb from the TSS of expressed genes in L1236 cells transduced with a non-targeting control shRNA. (**D**) A meta-gene plot showing mean BRG1 ChIP-seq coverage aligned between -3 kb to 3 kb from the TSS of expressed genes in L1236 cells transduced with H2A.B-targeting shRNA. ChIP-seq experiments were performed on H2A.B inducible knockdown L1236 cells collected on day 5 post-doxycycline induction. Lower panels show the corresponding heatmaps for all expressed genes.

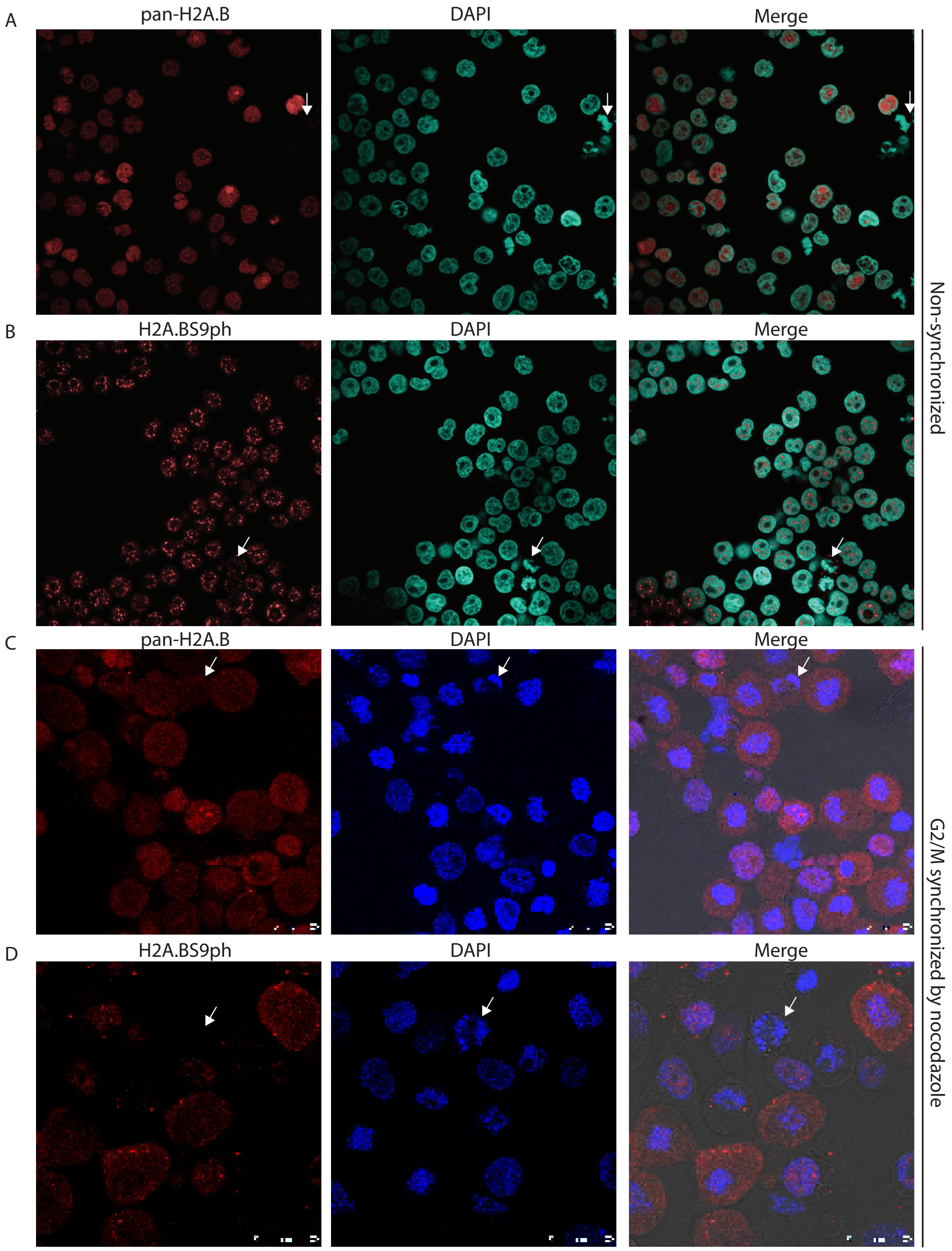
A
pan-H2A.B
DAPI
Merge
B
H2A.BS9ph
DAPI
Merge
C
pan-H2A.B
DAPI
Merge
D
H2A.BS9ph
DAPI
Merge
Non-synchronized
G2/M synchronized by nocodazole

Fig. S13. H2A.B and H2A.BS9ph are excluded from mitotic chromatin. (A) Representative confocal images of asynchronously growing L428 cells immunostained with anti-H2A.B and anti-H2A.BS9ph antibodies, and counterstained with DAPI. **(B)** Representative confocal images of L428 cells synchronized at the G2/M phase of the cell cycle using 100 ng/ml nocodazole for 24 hours and immunostained with anti-H2A.B and anti-H2A.BS9ph antibodies, and counterstained with DAPI. A representative mitotic cell in each image is indicated by a white arrow. H2A.B or H2A.BS9ph were not detected on mitotic chromosomes (N=120).

Sample	“Light” pull-down	“Medium” pull-down	“Heavy” pull-down
7F	Unmodified	R7me2a	R7me
7M	R7me	unmodified	R7me2a
7R	R7me2a	R7me	Unmodified
15F	Unmodified	n/a	R15me
15R	R15me	n/a	Unmodified
9F	Unmodified	n/a	S9ph
9R	S9ph	n/a	Unmodified

Table S1. List of biotinylated H2A.B N-terminal peptides used for SILAC-based streptavidin protein pull-downs. The differentially labelled proteins isolated by the unmodified and modified peptides were combined in equal proportions for subsequent LC-MS/MS analysis as shown.

Unmodified > R7me2a	R7me > R7me2a	UniProt accession	Function
SRSF6	SRSF6	Q13247	RNA splicing
SRSF5	n/a	Q13243	RNA splicing
TRA2B	TRA2B	P62995	RNA splicing
SNRNP40	SNRNP40	Q96DI7	RNA splicing
ALYREF	n/a	Q86V81	RNA splicing
NCBP2	n/a	P52298	RNA splicing
HNRNPK	n/a	P61978	RNA splicing
AKAP8L	AKAP8L	Q9ULX6	RNA splicing
n/a	HNRNPU	Q00839	RNA splicing
n/a	HNRNPD	Q14103	RNA splicing
n/a	SF3B4	Q15427	RNA splicing
n/a	NCBP1	Q09161	RNA splicing
n/a	CDC5L	Q99459	RNA splicing
n/a	ZCCHC8	Q6NZY4	RNA splicing
n/a	RPS19	P39019	Ribosomal subunit
n/a	RPS12	P25398	Ribosomal subunit
RPS28	n/a	P62857	Ribosomal subunit
n/a	ZCCHC3	Q9NUD5	Immune response
NAP1L1	n/a	P55209	Histone chaperone
n/a	QSER1	Q2KHR3	Unknown
n/a	KRI1	Q8N9T8	Unknown

Table S2. List of proteins that only bound to the unmodified or R7me peptide, but not to the R7me2a peptide.

Unmodified > R7me2a	R7me > R7me2a	UniProt accession	Function
YBX1	YBX1	P67809	RNA splicing
n/a	PABPC1	P11940	RNA splicing
RPS18	n/a	P62269	Ribosomal subunit
RPS19	n/a	P39019	Ribosomal subunit
RPS12	n/a	P25398	Ribosomal subunit
RPS5	RPS5	P46782	Ribosomal subunit
RPL11	n/a	P62913	Ribosomal subunit
RPL5	n/a	P46777	Ribosomal subunit
RPS16	n/a	P62249	Ribosomal subunit
BYSL	BYSL	Q13895	rRNA processing
LARP1	LARP1	Q6PKG0	mRNA translation regulation
n/a	HP1BP3	Q5SSJ5	Heterochromatin component

Table S3. The proteins with Log_2 forward ratio < -1 and Log_2 reverse ratio > 1, which were deemed as significant protein readers for the unmodified and R7me peptides (Fig.S6).

S9ph > unmodified	UniProt accession	Function
POLR1C	O15160	rRNA transcription
UBN2	Q6ZU65	Nucleosome assembly
NPAT	Q14207	Regulation of transcription
SMARCE1	Q969G3	SWI/SNF chromatin remodelling
RUVBL1	Q9Y265	ATPase and helicase
DBT	P11182	Branched-chain alpha-keto dehydrogenase complex
PRR11	Q96HE9	Cell cycle progression
LBR	Q14739	Nuclear envelope assembly

Table S4. List of proteins that only bound to the S9ph peptide, but not to the unmodified peptide.

S9ph > unmodified	UniProt accession	Function
POLR1A	O95602	rRNA transcription
TAF1D	Q9H5J8	rRNA transcription
TAF1C	Q15572	rRNA transcription
TTF1	Q15361	rRNA transcription
TCOF1	Q13428	rRNA transcription
UBN1	Q9NPG3	Nucleosome assembly
CABIN1	Q9Y6J0	Nucleosome assembly
HIRA	P54198	Nucleosome assembly
MYBBP1A	Q9BQG0	rRNA processing
NOM1	Q5C9Z4	rRNA processing
NAT10	Q9H0A0	rRNA processing
MTDH	Q86UE4	Regulation of transcription
BBX	Q8WY36	Regulation of transcription
PBRM1	Q86U86	SWI/SNF chromatin remodelling
RFC4	P35249	Replication
NOC3L	Q8WTT2	Replication
ATAD5	Q96QE3	DNA damage response
SGO2	Q562F6	Meiosis
CT45A3	Q8NHU0	Unknown
C1orf35	Q9BU76	Unknown
GPATCH4	Q5T3I0	Unknown

Table S5. The proteins with Log_2 forward ratio > 1 and Log_2 reverse ratio < -1, which were deemed as significant protein interactors for the S9ph modified peptides (Fig. S6)

R7me2a > unmodified	R7me2a > R7me	UniProt accession	Function
ARID2	ARID2	Q68CP9	SWI/SNF chromatin remodelling
SMARCC1	SMARCC1	Q92922	SWI/SNF chromatin remodelling
BRD9	n/a	Q9H8M2	SWI/SNF chromatin remodelling
n/a	BRD7	Q9NPI1	SWI/SNF chromatin remodelling
n/a	ACTL6A	O96019	SWI/SNF chromatin remodelling
ESF1	ESF1	Q9H501	Pre-rRNA processing
UTP20	n/a	O75691	Pre-rRNA processing
NPAT	NPAT	Q14207	Regulation of transcription
ZNF292	ZNF292	O60281	Regulation of transcription
KPNB1	n/a	Q14974	Protein transport
RUVBL2	RUVBL2	Q9Y230	ATPase and helicase
KDM5A	KDM5A	P29375	Histone demethylase
NSD1	n/a	Q96L73	Histone methyltransferase
SHMT2	SHMT2	P34897	Hydroxymethyltransferase
ATP5B	ATP5B	P06576	ATP synthase, mitochondrial
UBN2	UBN2	Q6ZU65	Nucleosome assembly
n/a	UBN1	Q9NPG3	Nucleosome assembly

Table S6. List of proteins that only bound to the R7me2a peptide, but not to the unmodified or R7me peptide.

R8me7a > unmodified	R7me2a > R7me	UniProt accession	Function
SMARCA2	n/a	P51531	SWI/SNF chromatin remodelling
SMARCC2	n/a	Q8TAQ2	SWI/SNF chromatin remodelling
n/a	PBRM1	Q86U86	SWI/SNF chromatin remodelling
NOM1	NOM1	Q5C9Z4	Pre-rRNA processing
RRP1B	RRP1B	Q14684	Pre-rRNA processing
MYBBP1A	MYBBP1A	Q9BQG0	Pre-rRNA processing
WDR18	n/a	Q9BV38	Pre-rRNA processing
LAS1L	n/a	Q9Y4W2	Pre-rRNA processing
LYAR	n/a	Q9NX58	Pre-rRNA processing
n/a	NAT10	Q9H0A0	Pre-rRNA processing
n/a	KNOP1	Q1ED39	Pre-rRNA processing
TAF1A	TAF1A	Q15573	rRNA transcription
TAF1C	TAF1C	Q15572	rRNA transcription
TAF1D	TAF1D	Q9H5J8	rRNA transcription
TTF1	TTF1	Q15361	rRNA transcription
TCOF1	TCOF1	Q13428	rRNA transcription
BBX	BBX	Q8WY36	Regulation of transcription
MTDH	MTDH	Q86UE4	Regulation of transcription
n/a	PRDM10	Q9NQV6	Regulation of transcription
MYO5A	MYO5A	Q9Y4I1	Vesicle transport
NOC3L	NOC3L	Q8WTT2	Replication
RFC4	RFC4	P35249	Replication
RUVBL1	RUVBL1	Q9Y265	ATPase and helicase
ATAD5	ATAD5	Q96QE3	DNA damage response
SGO2	n/a	Q562F6	Meiosis
SSBP1	SSBP1	Q04837	Mitochondrial biogenesis
HADHA	HADHA	P40939	Mitochondrial trifunctional protein
n/a	CABIN1	Q9Y6J0	Nucleosome assembly
n/a	CDCA2	Q69YH5	Phosphatase complex
GPATCH4	GPATCH4	Q5T3I0	Unknown
CT45A3	CT45A3	Q8NHU0	Unknown

Table S7. The proteins with Log_2 forward ratio > 1 and Log_2 reverse ratio < -1, which were deemed as significant protein interactors for the R7me2a modified peptides (Fig. S6).
